# *Peganum harmala* L.: Essential Oil Composition, Extraction Technologies, Biological Activities and Emerging Applications

**DOI:** 10.3390/molecules31183243

**Published:** 2026-09-14

**Authors:** Kawthar El Ahmadi, Khadija Haboubi, Sofia Zazouli, Oussama Khibech, Fahd A. Nasr, Ashraf Ahmed Qurtam, Joe Miantezila Basilua, Phuong Nguyen-Tri

**Affiliations:** 1 Laboratory of Engineering Sciences and Applications, National School of Applied Sciences of Al Hoceima, Abdelmalek Essâadi University, Al-Hoceima 32000, Morocco; k.haboubi@uae.ac.ma; 2 Department of Chemistry, Biochemistry and Physics, Université du Québec à Trois-Rivières, Trois-Rivières, QC G8Z 4M3, Canada; phuong.nguyen-tri@uqtr.ca; 3 Molecular Chemistry, Materials and Catalysis Laboratory, Faculty of Sciences and Techniques, Sultan Moulay Slimane University, B.P. 523, Beni-Mellal 23000, Morocco; sofia.zazouli@usms.ma; 4 Laboratory of Applied and Environmental Chemistry (LCAE), Department of Chemistry, Faculty of Sciences, Mohammed Premier University, Oujda 60000, Morocco; oussama.khibech.d24@ump.ac.ma; 5 Biology Department, College of Science, Imam Mohammad Ibn Saud Islamic University (IMSIU), Riyadh 11623, Saudi Arabia; aaqurtam@imamu.edu.sa; 6 Department of Biostatistics and Mathematics, Faculty of Pharmacy, Paris Cité University, 4, Avenue de l’Observatoire, 75006 Paris, France; joe.miantezila-basilua@u-paris.fr

**Keywords:** *Peganum harmala*, essential oil, β-carboline alkaloids, phytochemistry, green extraction, GC-MS, chemotyping, bioactivity, toxicological assessment, quality-by-design

## Abstract

*Peganum harmala* L. is a xerophytic medicinal and aromatic plant of considerable ethnopharmacological importance, characterized by a rich diversity of β-carboline alkaloids, phenolic compounds, and essential oil constituents. Despite increasing scientific interest, current knowledge remains fragmented across phytochemistry, essential oils, extraction technologies, biological activities, and safety assessments. This review provides an integrated overview of the essential oil composition, extraction methods, analytical characterization, biological properties, toxicological considerations, and emerging applications of *P. harmala*. Particular emphasis is given to organ-specific chemical variability, geographical and environmental influences on volatile profiles, and the importance of standardized chemical characterization for reliable bioactivity evaluation. Conventional extraction methods, including hydrodistillation and steam distillation, are critically compared with emerging green technologies such as ultrasound-assisted, microwave-assisted, and supercritical CO_2_ extraction. Advanced analytical tools, including GC-MS, LC-MS/MS, NMR, and chemometric approaches, are highlighted for comprehensive phytochemical profiling. Reported biological activities include antimicrobial, antifungal, antioxidant, anti-inflammatory, antidiabetic, anticancer, and agricultural effects, although their translation requires rigorous chemical standardization, mechanistic validation, and safety evaluation. Overall, this review emphasizes that the sustainable development of *P. harmala* should rely on quality-by-design strategies integrating botanical authentication, chemotyping, green extraction, and regulatory-oriented toxicological assessment.

## 1. Introduction

Medicinal and aromatic plants occupy a pivotal position in contemporary natural product research because they simultaneously represent biological resources, chemical libraries, cultural medicines, and industrial raw materials. Among them, *Peganum harmala* L. [1] is particularly instructive because it requires an integrated understanding of ethnomedicine, analytical chemistry, ecological adaptation, volatile chemistry, and toxicology rather than treating these disciplines as independent research domains [2]. The plant has traditionally been associated with ritual fumigation, gastrointestinal disorders, nervous system ailments, antiparasitic applications, and antimicrobial practices. Contemporary evidence, however, demonstrates that its pharmacological potential cannot be dissociated from its chemically diverse β-carboline alkaloids and highly variable volatile fraction [2,3]. Among the different phytochemical fractions, the essential oil has attracted increasing scientific interest owing to its chemical diversity, ecological functions, and expanding pharmaceutical, cosmetic, agricultural, and food-related applications [4].

Current research is evolving along two complementary directions. On one hand, phytochemical investigations continue to refine the characterization of seeds, leaves, roots, and fruits using advanced chromatographic and spectrometric techniques [5,6]. On the other hand, biological studies increasingly report antimicrobial, antioxidant, anti-inflammatory, enzyme-inhibitory, and cytotoxic activities. Nevertheless, a major limitation of the current literature is that many biological investigations remain insufficiently connected to standardized chemical markers, extraction yields, plant organ specificity, and geographical metadata [7,8]. Consequently, a critical review should move beyond simply cataloging biological activities and instead identify which chemical signatures are reproducible, which extraction strategies best preserve them, and which safety considerations must be addressed before translational development [9,10].

This review was designed to address these knowledge gaps by integrating essential-oil composition, extraction technologies, analytical characterization, biological activities, toxicological evidence, and emerging applications within a unified scientific framework [1,2]. Its objective is not to promote *P. harmala* as an unqualified therapeutic resource, but rather to define the scientific conditions under which it may be investigated as a source of functional molecules, pharmaceutical ingredients, agricultural bioprotectants, cosmetic ingredients, and formulation leads. Such an approach is particularly important for alkaloid-rich medicinal plants, where the same chemical characteristics responsible for potent biological activities may also determine toxicity, herb–drug interactions, and regulatory constraints.

Although several comprehensive reviews have previously summarized the phytochemistry, pharmacological properties, and toxicological aspects of *Peganum harmala* [11,12,13,14], most have focused on individual themes or specific biological activities. In contrast, the present review provides an integrated and updated perspective by combining phytochemical composition, extraction technologies, analytical methodologies, biological activities, toxicological evaluation, and translational considerations within a unified Quality-by-Design (QbD) framework. By explicitly linking raw material characteristics, extraction processes, phytochemical variability, biological activity, and safety, this review highlights how these interconnected factors can support the future standardization, quality control, and rational development of *P. harmala*-based pharmaceutical, nutraceutical, and botanical products.

### 1.1. Importance of Medicinal and Aromatic Plants

Medicinal and aromatic plants are no longer interpreted solely through the perspective of traditional medicine; they are increasingly recognized as highly complex chemical systems whose reproducibility depends on genetic background, plant organ, developmental stage, environmental conditions, harvesting practices, post-harvest handling, storage, and extraction methodology [15,16]. Essential oils constitute one of the most dynamic phytochemical fractions within these systems, comprising dozens to hundreds of low-molecular-weight volatile compounds whose composition may vary considerably according to ecological conditions and extraction parameters. Consequently, essential oil research requires rigorous botanical authentication together with robust analytical characterization [17,18].

The medicinal plant field has likewise evolved from descriptive bioactivity screening toward mechanism-oriented and chemically informed investigations. Antioxidant or antimicrobial activities are now considerably more informative when supported by comprehensive chemical fingerprints, mechanistic assays, appropriate controls, cytotoxicity evaluation, and reproducible batch documentation [19,20]. This requirement is particularly relevant for *P. harmala*, whose seed extracts are enriched in harmine and harmaline, whereas volatile fractions contain chemically distinct constituents whose contribution to biological activity depends strongly on the extraction method employed. Therefore, a comprehensive evaluation should explicitly connect extraction technology to chemical composition and chemical composition to biological function [21,22].

### 1.2. Peganum harmala L. as a Traditional Medicinal Species

*P. harmala* is a perennial, stress-tolerant species naturally adapted to arid and semi-arid ecosystems. Its ecological resilience, characteristic aroma, abundant seed production, and chemically mediated defense mechanisms have contributed both to its persistence in traditional medicine and to ecological discussions concerning allelopathy and invasiveness [21,22]. Traditional medicinal applications primarily involve seeds and fumigation practices, although roots, leaves, and aerial parts have also been widely documented. These ethnobotanical observations provide valuable hypotheses for scientific investigation but cannot substitute for rigorous evaluation of phytochemical composition, exposure, pharmacokinetics, pharmacodynamics, and toxicological safety [23,24].

The ethnopharmacological relevance of *P. harmala* is closely linked to its unique phytochemical architecture. Unlike many aromatic plants whose biological properties are largely determined by monoterpenes and sesquiterpenes, *P. harmala* combines volatile constituents with pharmacologically active nitrogen-containing β-carboline alkaloids [25,26]. This dual chemical profile complicates the interpretation of essential oil studies because biological responses may result from volatile metabolites, co-extracted semi-volatile compounds, alkaloid contamination, organ-specific chemistry, or methodological variability. Accordingly, the present review considers the essential oil as one component of a broader phytochemical system rather than as an isolated product [27,28].

### 1.3. Ethnopharmacological Significance

Ethnopharmacological evidence provides an important hypothesis-generating framework by identifying medicinal uses repeatedly recognized across different cultures; however, it should not be interpreted as direct evidence of efficacy [29]. In *P. harmala*, traditional practices suggest antimicrobial, antiparasitic, neuroactive, and anti-inflammatory properties, whereas contemporary investigations additionally report enzyme-inhibitory, cytotoxic, phytotoxic, and agricultural activities [30,31]. The principal scientific challenge is therefore to translate these heterogeneous observations into reproducible evidence by clearly defining plant organ, extract type, chemical markers, experimental model, biological endpoint, and safety margin [32,33].

A critical interpretation of ethnopharmacological knowledge also requires careful consideration of toxicological risk. Traditional fumigation or low-dose external applications cannot be directly extrapolated to concentrated extracts, purified alkaloids, or orally administered formulations [1,2]. Consequently, this review clearly distinguishes historical medicinal use from translational readiness and emphasizes that future product development should rely on standardized extraction procedures, comprehensive analytical quality control, dose-response investigations, mechanistic validation, and rigorous toxicological assessment [34]. This should not be regarded as a limitation of *P. harmala*, but rather as an essential prerequisite for scientifically credible and clinically relevant development [35,36].

### 1.4. Objectives and Scope of the Review

Accordingly, this review aims to summarize the botanical identity, taxonomy, and geographical distribution of *Peganum harmala*; integrate current knowledge on its β-carboline alkaloids, phenolic compounds, flavonoids, and volatile constituents [1,2]; critically compare conventional and emerging extraction technologies with particular emphasis on essential oil yield, selectivity, sustainability, and environmental performance; interpret reported biological activities through mechanism-oriented and chemically supported evidence rather than descriptive activity screening alone [11,12]; and identify the current knowledge gaps and future research priorities required for the responsible development of pharmaceutical, cosmetic, food preservation, agricultural, and other industrial applications [13,14].

## 2. Literature Search Strategy

This review was conducted as a narrative review to provide a comprehensive overview of the current knowledge on *Peganum harmala*. The literature was retrieved from major scientific databases, including PubMed, Scopus, Web of Science, ScienceDirect, and Google Scholar. Publications available up to July 2026 were considered using combinations of keywords such as “*Peganum harmala*”, “harmine”, “harmaline”, “β-carboline alkaloids”, “essential oil”, “phytochemistry”, “extraction”, “biological activities”, “toxicity”, “Quality-by-Design”, and “translational perspectives”. Priority was given to peer-reviewed original research articles and recent review papers reporting phytochemical composition, extraction technologies, biological activities, toxicological aspects, analytical methodologies, and quality-related studies on *Peganum harmala*. Additional relevant publications were identified through manual screening of the reference lists of the selected articles to ensure comprehensive coverage of the available literature.

## 3. Methodology and Evidence Synthesis Strategy

**Figure 1:**
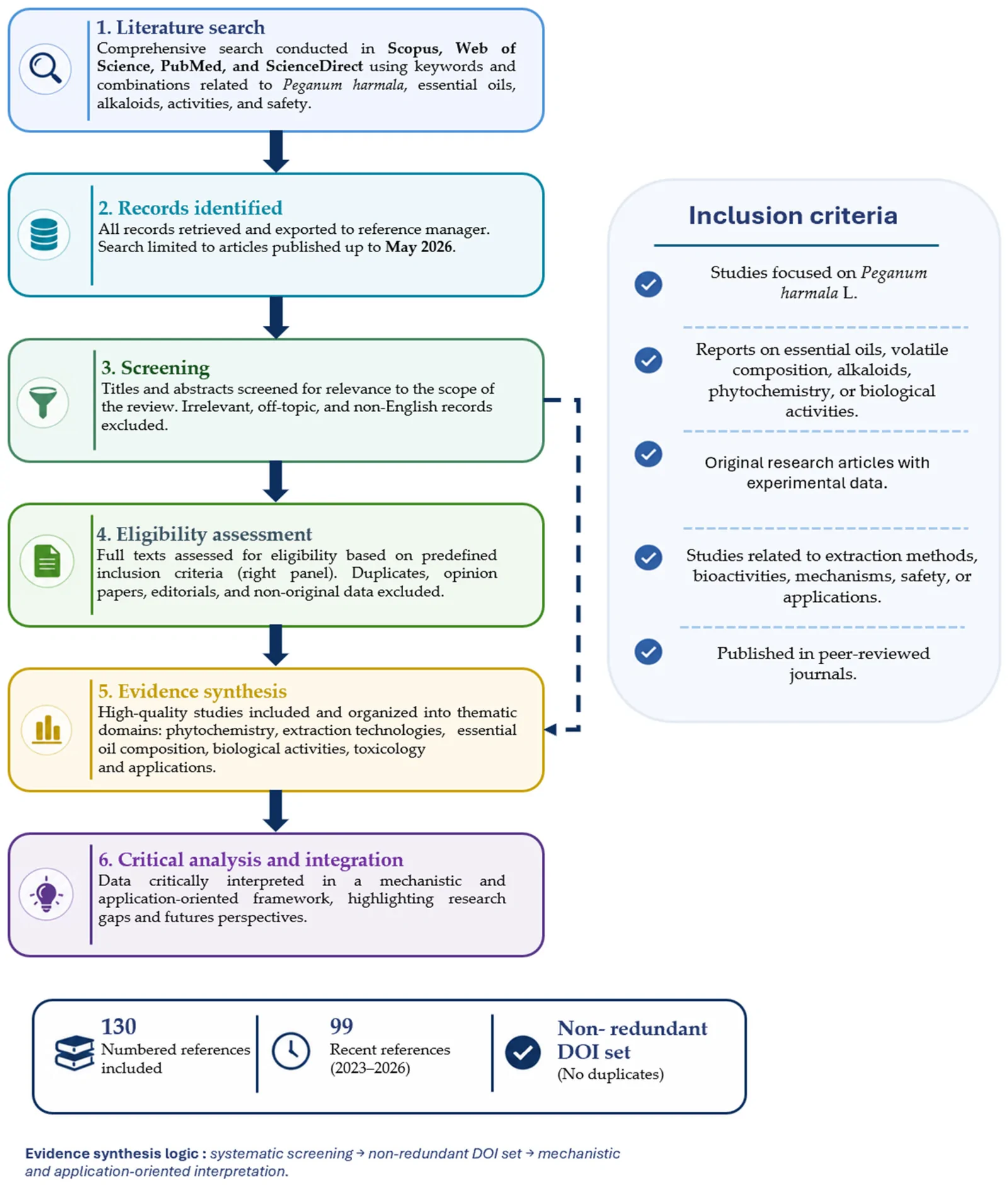
Structured evidence workflow used to organize the *Peganum harmala* literature and maintain non-redundant DOI traceability [11,12,13,14,15,16,17,18,19,20].

The review followed a structured evidence synthesis strategy based on transparent literature search, screening, eligibility assessment, and evidence integration, as illustrated in Figure 1. Searches were conceptually organized around *P. harmala*, harmine, harmaline, harmalol, harmane, essential oil, volatile compounds, GC-MS, extraction, hydrodistillation, ultrasound-assisted extraction, microwave-assisted extraction, supercritical carbon dioxide extraction, antioxidant, antimicrobial, antifungal, anti-inflammatory, antidiabetic, anticancer, toxicology, and application-related terms [11,12,13]. Priority was given to studies reporting explicit botanical authentication, plant organ specification, extraction methodology, analytical characterization, and DOI traceability [14].

Inclusion emphasized peer-reviewed research articles, reviews, and methodological studies relevant to *P. harmala* phytochemistry, essential oils, extraction technologies, analytical characterization, biological activities, toxicology, and formulation science [11,12]. Exclusion criteria included duplicated DOI records, non-traceable references, publications lacking sufficient methodological information, and studies whose biological conclusions were not supported by extract characterization or chemical evidence. As summarized in Figure 1, the final bibliography was organized to ensure a logical progression from general background and methodology to botany, phytochemistry, extraction technologies, analytical characterization, biological activities, toxicology, applications, and future research directions [15,16].

Because the available literature spans multiple scientific disciplines and experimental designs, evidence was not statistically pooled. Instead, the review adopts a narrative evidence synthesis approach supported by comparative tables, conceptual figures, and an annotated evidence map [17]. This strategy is particularly appropriate for heterogeneous datasets differing in plant organ, extraction protocol, analytical platform, biological assay, and reporting quality [18,19]. Rather than estimating a single pooled effect size, the objective is to identify areas of convergence and divergence, highlight methodological limitations, and define key knowledge gaps that should guide future research on *P. harmala* [20].

The structured workflow presented in Figure 1 summarizes the overall strategy adopted for literature identification, screening, eligibility assessment, and evidence synthesis. To further improve the transparency and reproducibility of the review process, the inclusion and exclusion criteria used during literature evaluation are summarized in Table 1. Particular emphasis was placed on botanical authentication, comprehensive phytochemical characterization, essential oil profiling, extraction methodology, biological evidence, toxicological evaluation, and methodological quality to ensure that the synthesized evidence accurately reflects the current scientific understanding of *Peganum harmala*.

## 4. Botanical Description and Geographical Distribution

### 4.1. Taxonomy

*Peganum harmala* L. is a perennial xerophytic herb belonging to the genus *Peganum*. Although it was historically classified within the family Zygophyllaceae, recent molecular phylogenetic studies support its placement in the family Nitrariaceae [1]. The genus *Peganum* comprises only a few recognized species, among which *P. harmala* is the most widely distributed and extensively investigated due to its remarkable accumulation of β-carboline alkaloids [2]. Morphologically, the species is distinguished by its deeply divided linear leaves, solitary white flowers with five petals, globose capsules, and numerous angular dark-brown seeds, which constitute the principal medicinally used plant organ [3].

### 4.2. Botanical Characteristics

Botanically, *P. harmala* is adapted to dry, disturbed and saline-prone environments. Its morphology includes deeply divided leaves, solitary flowers, capsule fruits and small angular seeds that are widely used in traditional practices [21,22]. The plant’s ecological toughness is relevant to chemistry because stress-tolerant species often invest in chemical defense, allelopathy and secondary metabolites that can deter herbivores, microbes and competing plants. This ecological logic helps explain the coexistence of volatile constituents, phenolics and bioactive beta-carbolines [24,25].

The seed is the most studied organ, largely because it is accessible, culturally important and alkaloid-rich [5]. However, leaves and roots deserve more systematic investigation for essential oil and volatile signatures [6]. Organ-specific profiling is crucial because the seed-dominant literature can distort expectations for aerial-part oils and because industrial applications may prefer leaves or aerial biomass if seed harvesting is limited, seasonal or ecologically undesirable [7,8].

### 4.3. Geographical Distribution

The natural and introduced distribution of *P. harmala* spans arid and semi-arid regions across North Africa, the Mediterranean basin, the Middle East, Central Asia and parts of Asia [21,22]. The plant’s occurrence in disturbed habitats, roadsides, rangelands and marginal soil has generated interest not only in medicinal use but also in ecological management [26]. For chemical studies, geography must be treated as an experimental factor because climate, soil chemistry, water availability and altitude may influence volatile biosynthesis and alkaloid accumulation [27].

### 4.4. Traditional Uses in Different Regions

Traditional uses of *P. harmala* vary considerably across its geographical distribution, reflecting the cultural and medicinal practices of different regions. In North Africa, particularly in Morocco and Algeria, the seeds are traditionally burned as fumigants to ward off the evil eye and are also used for digestive disorders, rheumatic pain, and skin diseases [1]. In the Middle East, including Iran and Iraq, *P. harmala* has long been employed in traditional medicine for its antimicrobial, antiparasitic, analgesic, and anti-inflammatory properties, while ritual fumigation remains a common cultural practice [2]. In South Asia, especially in Pakistan and India, the plant is incorporated into Unani and traditional medicine to manage gastrointestinal disorders, diabetes, hypertension, and certain neurological conditions [3]. Despite these regional differences, the seeds remain the most frequently used plant part, administered as fumigants, decoctions, powders, or topical preparations depending on the intended therapeutic application [37].

The botanical, ecological, and ethnopharmacological variables discussed throughout this section directly influence the phytochemical composition, essential oil profile, and biological activities of *P. harmala*. To improve reproducibility and facilitate meaningful comparisons among future studies, the principal variables that should be systematically reported are summarized in Table 2.

## 5. Phytochemical Composition of *Peganum harmala*

### 5.1. Alkaloids

Beta-carboline alkaloids are the central chemical signature of *P. harmala* [33]. Harmine, harmaline, harmalol, harmol, harmane and related derivatives form a structurally coherent but pharmacologically diverse class [39]. Their planar heteroaromatic systems, basic nitrogen atoms and redox-active features allow interactions with enzymes, nucleic acids, transporters and receptors [40,41]. This explains why *P. harmala* extracts can display strong biological effects even when total extract concentrations are low [42]. β-carboline alkaloids originate primarily from the condensation of tryptamine-derived intermediates through the Pictet–Spengler reaction, followed by a series of oxidation and methylation steps leading to structurally diverse derivatives such as harmine, harmaline, harmalol, and harmane. This common biosynthetic origin explains their close structural relationship while also accounting for the differences in their physicochemical properties and biological activities [36]. Beyond their pharmacological importance, β-carboline alkaloids are believed to contribute to the ecological fitness of *P. harmala* by protecting the plant against herbivores, microbial pathogens, and environmental stress, thereby supporting its adaptation to arid and semi-arid ecosystems [37].

The alkaloid profile is not static. It depends on organ, maturity, geography, extraction solvent, pH, processing and analytical detection strategy [33,39]. Seeds are often prioritized because they contain substantial beta-carbolines, but roots and fruits can provide structurally informative analogues [43]. New alkaloids reported in recent work demonstrate that the chemical inventory remains incomplete, particularly when high-resolution mass spectrometry and bioactivity-guided isolation are combined [44].

#### 5.1.1. Harmine

Harmine is among the most intensively studied *P. harmala* alkaloids and is mechanistically important because of reported enzyme inhibition, anticancer signaling effects and neuropharmacological relevance [42,45]. In terms of chemical standardization, harmine should be treated as both a bioactive molecule and a safety marker [46]. Its presence can support mechanistic interpretation, but it also requires attention to dose, route, monoamine oxidase interactions and potential herb–drug interactions [47,48].

#### 5.1.2. Harmaline

Harmaline is structurally related to harmine but differs in saturation state and pharmacological behavior [42]. Its contribution to extract activity cannot be assumed from harmine concentration alone [49]. Analytical methods should quantify harmaline separately, especially when extracts are intended for antimicrobial, enzyme-inhibitory or neuroactive screening [44]. Because harmaline-rich preparations may generate strong biological signals, they require careful cytotoxicity and safety interpretation [35,36].

#### 5.1.3. Harmalol

Harmalol and related hydroxylated beta-carbolines are important because polarity and hydroxylation can change solubility, redox behavior, metabolism and chromatographic detectability [39]. Their presence underscores the need for multi-platform analysis [42]. A GC-MS-only workflow may describe volatile fractions well but cannot fully resolve polar alkaloids, while LC-MS/MS and HPLC-DAD can provide more reliable quantitation of these nitrogen-containing constituents [50,51,52].

#### 5.1.4. Harmane

Harmane illustrates the boundary between plant phytochemistry and broader beta-carboline exposure science. It can occur in botanical matrices and in other contexts linked with processing and combustion [37]. In *P. harmala* research, harmane should be interpreted together with harmine, harmaline and harmalol rather than as an isolated marker [35]. This is particularly relevant for studies of smoke-derived volatile and traditional fumigation [53,54].

### 5.2. Phenolic Compounds

#### 5.2.1. Phenolic Acids

Although beta-carbolines dominate much of the *P. harmala* literature, phenolic compounds are indispensable for interpreting antioxidant and anti-inflammatory signals [4]. Phenolics can contribute hydrogen atom transfer, electron transfer capacity, metal chelation behavior and modulation of oxidative signaling pathways [55,56]. Without phenolic quantitation, total antioxidant capacity is difficult to attribute mechanistically, and alkaloid-rich extracts may be overcredited for effects partly driven by non-alkaloid constituents [57].

Phenolic profiles should be reported alongside extraction polarity. Hydroalcoholic, aqueous and organic extracts enrich different chemical families, and comparisons across studies are weak when solvent systems are not specified [16,19]. Future *P. harmala* research should integrate total phenolic content, targeted LC-MS/MS of major phenolics and untargeted metabolomics, then relate these features to biological assays by correlation or multivariate modeling [51,58].

#### 5.2.2. Flavonoids

Flavonoids in *P. harmala* have received less attention than beta-carbolines, but they are important because they may modulate antioxidant, antimicrobial and anti-inflammatory outcomes and influence extract polarity [4,55,56]. The common practice of reporting total flavonoid content without structural assignment should be regarded as preliminary. For mechanistic interpretation, studies should resolve individual flavonoids, glycosylation patterns and co-occurrence with phenolics and alkaloids [4,55,56,57].

### 5.3. Essential Oils and Volatile Constituents

The volatile fraction of *P. harmala* is particularly sensitive to extraction strategy. Hydrodistillation, HS-SPME, solvent extraction and headspace methods may emphasize different chemical windows [5,6]. Recent seed volatilome work has shown that extraction method can substantially alter the compounds detected, which has direct implications for essential oil interpretation, network pharmacology, antimicrobial testing and product standardization. Therefore, the phrase ‘*P. harmala* essential oil’ should always be linked to extraction conditions [7,8,9].

Volatile profiles reported in the literature include terpenoid, oxygenated, aromatic and nitrogen-containing constituents, but the relative dominance of these classes may change by geography, organ and method [5,8]. Studies should distinguish true essential oils obtained by distillation from headspace volatiles, smoke condensates and solvent-extracted volatile-rich fractions [15,16]. Mixing these categories can generate misleading comparisons of yield, composition and biological activity [37].

### 5.4. Chemical Variability Among Plant Organs

Organ specificity is one of the most important unresolved dimensions of *P. harmala* chemistry [5,6]. Seeds provide abundant alkaloid information, while leaves are relevant for essential oil, aerial biomass valorization and sustainable harvesting [8]. Roots may concentrate distinctive alkaloids and ecological signals, but destructive harvesting raises sustainability issues. Fruits and smoke-derived fractions add further chemical complexity [33,39]. A mature field will require organ-resolved chemotype maps rather than extrapolation from seeds to the whole plant [37].

The organ-specific chemical characteristics discussed above have important implications for phytochemical investigation and analytical method selection. As summarized in Table 3, each plant organ requires dedicated analytical strategies and cautious interpretation to avoid inappropriate extrapolation of chemical and biological data.

The organ-specific phytochemical characteristics summarized in Table 3 highlight the importance of considering each plant organ as a distinct chemical entity rather than extrapolating results from seeds to the entire plant. This variability has important implications for phytochemical characterization, biological evaluation, and quality control procedures, emphasizing the need for organ-specific sampling and appropriate analytical strategies in future studies.

### 5.5. Essential Oil Characterization

#### 5.5.1. Essential Oil Yield

Essential oil yield in *P. harmala* is best interpreted as a process variable rather than a fixed species trait [5,6,7]. Yield depends on organ, drying conditions, biomass particle size, water content, distillation time and extraction technique [8,9]. A low yield may still be valuable if the oil contains distinctive functional markers, whereas a high yield may be less useful if dominated by degradation products or poorly reproducible constituents. Therefore, yield should be reported together with chemical profile and bioactivity retention [59,60].

#### 5.5.2. Chemical Composition Determined by GC-MS

GC-MS remains the central analytical tool for *P. harmala* volatile and essential oil characterization [15,16]. However, high-quality GC-MS reporting requires more than a compound list: it should provide retention indices, spectral match criteria, reference libraries, standards where available, column type, temperature program, injection conditions and relative abundance calculations. Without these details, interstudy comparisons become fragile and minor compounds may be overinterpreted [50,61].

Recent analytical developments suggest that *P. harmala* studies should combine targeted and untargeted approaches [51,62]. GC-MS can profile volatiles, LC-MS/MS can quantify alkaloids and phenolics, NMR can support structural confirmation and quantitative quality control, while molecular networking can organize unknown features into chemical families [63,64]. This multi-platform strategy is essential because the plant does not fit neatly into a single analytical category [58,65,66,67,68].

#### 5.5.3. Major Volatile Constituents

The major volatile constituents reported for *P. harmala* vary across extraction methods and plant materials, reinforcing the need for cautious interpretation [5,6]. Studies using hydrodistillation, HS-SPME or other volatile capture strategies may detect different profiles because each method samples a different chemical window [7]. Major constituents should therefore be discussed as method-specific markers rather than universal plant signatures unless replicated across organ, geography and extraction conditions [8,9].

#### 5.5.4. Influence of Geographical Origin

Geographical origin may influence the volatile profile through climate, soil, altitude, water availability, photoperiod and local stress regimes. However, geography can be confounded by harvest stage, drying and extraction [21,22]. A rigorous geographical study would use replicated collections, standardized processing, multiple organs and multivariate analysis to separate environmental effects from methodological variation. Such work could identify chemotypes suitable for targeted industrial or pharmacological exploration [38,61].

#### 5.5.5. Influence of Environmental and Agronomic Factors

Environmental and agronomic factors remain underexplored in *P. harmala* compared with other aromatic crops. Soil salinity, drought, temperature, nutrient status, plant density and disturbance may influence metabolite allocation and volatile profiles [21,22]. Because *P. harmala* often grows in marginal environments, it provides a useful model for stress-associated secondary metabolism. Future experiments should move from opportunistic collection toward controlled cultivation and stress factor manipulation [25,26].

#### 5.5.6. Critical Comparison of Analytical Approaches

Comparative analysis of the available literature indicates considerable variability in the quality and completeness of phytochemical investigations. The most robust studies integrate accurate botanical identification, standardized extraction procedures, comprehensive chemical characterization, and biologically relevant assays, whereas others report either biological activity without sufficient chemical evidence or phytochemical data without functional validation [11,15,16]. These observations highlight the need for standardized analytical workflows and harmonized reporting practices to improve the reproducibility and comparability of future studies.

Given the chemical complexity of *P. harmala*, no single analytical platform can comprehensively characterize all metabolite classes. Therefore, complementary analytical techniques are often required. The principal analytical methods currently employed for phytochemical characterization, together with their major applications, strengths, and limitations, are summarized in Table 4.

## 6. Extraction Technologies

### 6.1. Hydrodistillation

Hydrodistillation remains the reference technique for essential oil recovery because it is accessible, historically established and compatible with many pharmacognostic laboratories [8,9]. For *P. harmala*, however, hydrodistillation must be interpreted carefully because high temperature and prolonged contact with water can alter thermolabile constituents, promote hydrolysis, and underrepresent highly volatile or water-soluble compounds. Yield alone is therefore insufficient; compositional integrity and marker recovery must also be evaluated [59,70].

A high-quality hydrodistillation report should include plant organs, dry mass, moisture content, particle size, water-to-plant ratio, distillation time, condenser conditions, oil separation method, storage temperature and analytical timing [15,16]. Without these parameters, comparisons across studies are weak and apparent geographical differences may actually reflect processing variability [71]. For *P. harmala*, such reporting is especially important because alkaloid-rich matrices can complicate volatile extraction and interpretation [50].

### 6.2. Steam Distillation

Steam distillation can reduce direct plant–water contact and may be preferable when large-scale volatile recovery is desired. Its value for *P. harmala* should be tested through direct comparison with hydrodistillation under matched biomass conditions [59,70]. Important evaluation criteria include oil yield, relative abundance of oxygenated constituents, degradation markers, energy consumption, scalability and biological activity retention. Steam distillation should not be assumed superior without paired chemical fingerprints [71,72].

### 6.3. Solvent Extraction

Solvent extraction is powerful for recovering alkaloids, phenolics and semi-volatile constituents, but it produces chemically broader extracts rather than essential oils in the strict distillation sense [42]. This distinction is critical for *P. harmala* because solvent extracts may contain high levels of beta-carbolines that dominate bioactivity and toxicity [55,73]. Studies should therefore avoid describing solvent extracts as essential oils unless the product is truly volatile oil isolated by an accepted method [71].

### 6.4. Ultrasound-Assisted Extraction

Ultrasound-assisted extraction can improve mass transfer by cavitation, cell wall disruption and accelerated solvent penetration [17,19]. For *P. harmala*, UAE is attractive for phenolics, alkaloids and possibly volatile-rich solvent extracts, but it requires optimization to avoid uncontrolled heating and degradation [70]. Its green value depends on solvent choice, time, energy input and the ability to achieve comparable marker recovery with lower environmental burden [74].

The most rigorous UAE studies would use design-of-experiments models integrating solvent composition, amplitude, temperature, extraction time, solid-to-liquid ratio and particle size [16,17]. Response variables should include total yield, harmine/harmaline recovery, volatile markers, phenolic content, antioxidant capacity and cytotoxicity [75,76,77,78]. Such integrated optimization would allow *P. harmala* extracts to be tailored to intended applications rather than generated by inherited protocols [79].

### 6.5. Microwave-Assisted Extraction

Microwave-assisted extraction and microwave-assisted hydrodistillation are attractive because they can reduce extraction time and energy demand while intensifying intracellular heating [17,20]. In aromatic plants, microwave approaches can modify both yield and compound ratios. For *P. harmala*, available evidence indicates that MAE represents a promising alternative extraction technique; however, its application to *P. harmala* remains comparatively limited. Because β-carbolines, phenolics, and volatile constituents may respond differently to microwave irradiation, the available data should be interpreted cautiously [70,80].

### 6.6. Supercritical Carbon Dioxide Extraction

Supercritical carbon dioxide extraction offers solvent-free or low-solvent recovery with tunable selectivity through pressure, temperature and co-solvent modulation [18,70]. It is particularly relevant when the goal is to avoid thermal degradation or residual organic solvents. For *P. harmala*, SC-CO_2_ has considerable potential to separate lipophilic, volatile, and semi-volatile fractions from alkaloid-rich polar fractions, although its practical performance depends on extraction conditions, process economics, and validated marker recovery [71,79].

### 6.7. Advantages and Limitations of Extraction Methods

No extraction method is universally optimal for *P. harmala* [5]. Hydrodistillation provides comparability with older essential oil studies; HS-SPME provides sensitive volatile fingerprints without bulk oil recovery; solvent extraction enriches alkaloids and phenolics; UAE and MAE can increase efficiency; SC-CO_2_ can improve selectivity and environmental profile [17,18]. The correct choice depends on the research question: volatile characterization, bioactivity screening, industrial scaling, formulation, or safety assessment [70,71].

**Figure 2:**
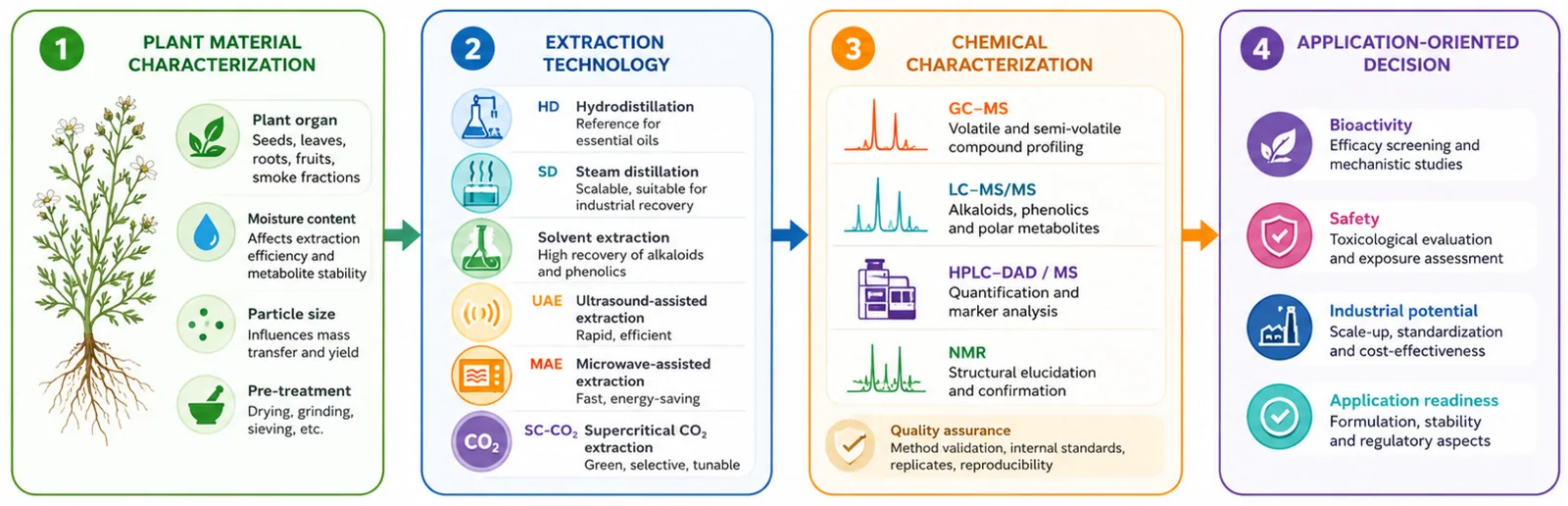
Analytical decision framework for *Peganum harmala* L. extraction.

The relationship between plant material characteristics, extraction technologies, analytical characterization, and application-oriented decision-making is summarized in Figure 2.

Although each extraction technique offers specific advantages, no single method is universally suitable for all classes of *P. harmala* phytochemicals. The selection of an appropriate extraction strategy should therefore consider the target metabolites, analytical objectives, extraction efficiency, environmental sustainability, and intended application. A comparative overview of the principal extraction technologies, together with their strengths, limitations, and recommended applications, is presented in Table 5.

## 7. Biological Activities and Mechanisms of Action

### 7.1. Antioxidant Activity

The antioxidant activity of *Peganum harmala* has been consistently demonstrated in numerous studies, although its interpretation requires careful consideration of both the experimental model and the phytochemical composition of the tested extracts [4]. Conventional chemical assays, including DPPH, ABTS, FRAP, and total antioxidant capacity (TAC), evaluate distinct mechanisms of antioxidant action and therefore should not be considered interchangeable indicators of antioxidant potential [55]. The antioxidant properties of *P. harmala* are mainly attributed to the combined action of phenolic compounds, flavonoids, and β-carboline alkaloids, which exert complementary radical-scavenging, metal-chelating, and redox-modulating activities [57,81].

Recent quantitative evidence demonstrates that extraction conditions strongly influence antioxidant efficacy. Khalid et al. reported that a 50% ethanolic extract exhibited significantly greater antioxidant activity (411.8 ± 3.5 μmol Trolox equivalents/g extract) than the absolute ethanolic extract (312.9 ± 8.2 μmol Trolox equivalents/g extract) [82,83]. This improvement was accompanied by markedly higher total phenolic (215.8 ± 3.5 vs. 155.8 ± 2.9 mg GAE/g extract) and flavonoid contents (112.1 ± 3.1 vs. 92.3 ± 1.8 mg catechin equivalents/g extract), highlighting the close relationship between phytochemical composition and antioxidant capacity. These findings further emphasize that solvent polarity is a critical determinant of bioactive compound recovery and, consequently, antioxidant performance [84].

Beyond cell-free chemical assays, in vivo evidence further supports the antioxidant potential of *P. harmala* [85,86,87]. In an aluminum chloride-induced Alzheimer’s disease model, treatment with a standardized methanolic seed extract significantly reduced hippocampal lipid peroxidation by decreasing malondialdehyde (MDA) levels by approximately 50%, while simultaneously increasing the expression of the antioxidant regulator Nrf2 by 1.8-fold and restoring glutathione (GSH) levels by approximately 2-fold compared with untreated animals. These findings indicate that the antioxidant effects of *P. harmala* extend beyond direct free radical scavenging and involve activation of endogenous antioxidant defense pathways, thereby contributing to its neuroprotective properties [88].

Despite these promising findings, direct comparison among published studies remains challenging because of substantial methodological heterogeneity. Variations in plant origin, harvested organ, extraction solvent, extraction procedure, phytochemical standardization, and antioxidant assays frequently lead to inconsistent results. Furthermore, many investigations rely exclusively on chemical antioxidant tests without confirming whether the observed activity translates into biologically relevant protection against oxidative stress in cellular or in vivo models. Consequently, antioxidant claims should be interpreted cautiously unless supported by comprehensive phytochemical characterization and complementary biological validation.

Future studies should therefore integrate multiple chemical assays with cell-based and in vivo oxidative stress models while correlating biological responses with quantitative phytochemical profiling using validated analytical techniques. Such standardized approaches would substantially improve the comparability of published studies and facilitate the identification of the principal constituents responsible for the antioxidant effects of *P. harmala*.

### 7.2. Antibacterial Activity

Based on the available evidence, the following recommendations are proposed by the authors to improve the reproducibility and comparability of future antimicrobial studies. These recommendations should be considered as evidence-informed perspectives rather than established consensus guidelines.

The antimicrobial activity of *Peganum harmala* has been extensively investigated against a broad spectrum of Gram-positive and Gram-negative bacteria, yeasts, and filamentous fungi, making it one of the most extensively documented biological properties of the species [73]. Antimicrobial activity has been reported for crude extracts, alkaloid-rich fractions, isolated β-carboline alkaloids, and essential oils, although the magnitude of the activity varies considerably depending on the plant organ, extraction procedure, chemical composition, microbial strain, and experimental protocol [57,89,90]. The antimicrobial effects are generally attributed to the combined action of β-carboline alkaloids, particularly harmine and harmaline, together with volatile terpenoids and phenolic constituents, which may interfere with microbial membrane integrity, nucleic acid synthesis, enzymatic activity, and intracellular redox homeostasis [57,89,90].

Recent quantitative studies further support the antimicrobial potential of *P. harmala*. Khalid et al. demonstrated that a hydroethanolic extract exhibited marked antibacterial activity against several pathogenic bacteria, producing inhibition zones of approximately 20 mm against *Staphylococcus aureus* and *Streptococcus* spp., and 15 mm against *Escherichia coli*. The higher antibacterial activity was associated with increased phenolic and flavonoid contents, suggesting that these compounds contribute synergistically with β-carboline alkaloids to the overall antimicrobial effect [84].

The antimicrobial efficacy of *P. harmala* is likely multifactorial rather than attributable to a single class of metabolites. β-carboline alkaloids have been shown to interfere with DNA replication, topoisomerase activity, monoamine oxidase inhibition, and other enzyme-mediated cellular processes, whereas essential oil constituents primarily disrupt membrane permeability, alter membrane fluidity, and induce oxidative imbalance within microbial cells. Such complementary mechanisms may explain the broad antimicrobial spectrum reported for crude extracts compared with isolated compounds. Nevertheless, convincing evidence of synergistic interactions remains limited because relatively few studies have employed checkerboard assays, fractional inhibitory concentration (FIC) indices, or time-kill analyses to quantify combination effects.

Another important limitation is that a large proportion of the published literature relies on crude extracts or only partially characterized fractions, making it difficult to identify the compounds responsible for the observed antimicrobial effects. Furthermore, microbiological evaluations are frequently restricted to a limited number of reference strains without comprehensive assessment against clinically relevant or multidrug-resistant isolates, thereby limiting the comparability and translational relevance of the available evidence.

Despite encouraging experimental results, the current body of evidence remains difficult to compare because of substantial methodological heterogeneity. Many studies use agar diffusion assays as the primary evaluation method without determining minimum inhibitory concentration (MIC) or minimum bactericidal concentration (MBC) values, while others lack appropriate solvent controls, reference antibiotics, standardized inoculum densities, or detailed phytochemical characterization of the tested extracts [91]. Consequently, inhibition zone diameters alone should not be interpreted as definitive indicators of antimicrobial potency, particularly when comparing extracts with different diffusion properties.

Future investigations should therefore adopt internationally standardized broth microdilution protocols (CLSI or EUCAST guidelines), systematically determine MIC and MBC values, include clinically relevant multidrug-resistant isolates, and correlate antimicrobial activity with comprehensive phytochemical profiling [89,90,92]. In addition, synergistic interactions between β-carboline alkaloids and essential oil constituents should be evaluated using checkerboard or time-kill methodologies, while formulation studies should further investigate the stability and vapor-phase antimicrobial activity of *P. harmala* essential oils for potential pharmaceutical and food preservation applications [84,93].

### 7.3. Antifungal Activity

The antifungal activity of *Peganum harmala* has attracted considerable interest because of its potential applications in the treatment of fungal infections, agriculture, and food preservation [57,89]. Various extracts, alkaloid-rich fractions, and essential oils have demonstrated inhibitory effects against clinically relevant yeasts and filamentous fungi, although the reported efficacy varies according to the plant organ, extraction method, chemical composition, fungal species, and experimental conditions [90,92]. β-carboline alkaloids, particularly harmine and harmaline, together with volatile terpenoids and phenolic constituents, are considered the principal compounds responsible for the antifungal activity of the plant.

Recent studies have expanded the understanding of the antifungal potential of *P. harmala* beyond conventional susceptibility assays by investigating its activity against fungal biofilms. Erfaninejad et al. evaluated the antibiofilm activity of a hydroethanolic seed extract against 33 clinical isolates of *Candida albicans* and reported complete biofilm eradication in 15 isolates, with minimum biofilm eradication concentration (MBEC) values ranging from 0.49 to 7.8 μg/mL. Notably, the lowest MBEC (0.49 μg/mL) was observed for isolate H043, whereas the highest value (7.8 μg/mL) was recorded for isolate H025, highlighting differences in susceptibility among clinical isolates [94].

Mechanistic investigations further demonstrated that treatment with *P. harmala* extract significantly downregulated the oxidative stress-related CAT1 gene (fold change = 0.038, *p* = 0.0068), while no statistically significant changes were observed for the biofilm regulatory genes EFG1 (fold change = 0.922, *p* = 0.5754) and BCR1 (fold change = 0.872, *p* = 0.529). These findings suggest that the antibiofilm activity of *P. harmala* is primarily associated with disruption of fungal oxidative stress homeostasis rather than direct modulation of biofilm regulatory pathways. Reduced CAT1 expression is expected to impair catalase-mediated detoxification of hydrogen peroxide, promoting intracellular reactive oxygen species accumulation and ultimately contributing to biofilm eradication [94].

Despite these promising findings, the available evidence remains limited by substantial methodological heterogeneity. Most studies have focused on *Candida albicans*, while other clinically important yeasts and filamentous fungi remain poorly investigated. In addition, differences in extraction procedures, phytochemical composition, inoculum preparation, susceptibility testing methods, and endpoint determination complicate direct comparisons among studies. Standardized evaluation of minimum inhibitory concentration (MIC), minimum fungicidal concentration (MFC), and biofilm-related parameters is therefore required to establish the true antifungal potential of *P. harmala*.

Future research should combine standardized antifungal susceptibility testing with comprehensive phytochemical characterization and mechanistic investigations to clarify the contribution of individual β-carboline alkaloids and their possible synergistic interactions with other phytochemicals. Moreover, evaluating the activity of *P. harmala* against multidrug-resistant fungal pathogens and mature biofilms will be essential to determine its translational potential for pharmaceutical, agricultural, and food preservation applications.

### 7.4. Anti-Inflammatory Activity

The anti-inflammatory potential of *Peganum harmala* is biologically plausible because β-carboline alkaloids and phenolic constituents can modulate oxidative signaling, enzyme activity, and cytokine-related pathways [55,95]. Harmaline and related constituents have been linked to lipoxygenase- and prostaglandin-mediated mechanisms, whereas crude extracts may influence nitric oxide production and other inflammatory mediators. However, in vitro anti-inflammatory effects should always be interpreted alongside cytotoxicity data, as an apparent reduction in inflammatory mediators may result from decreased cell viability rather than a specific pharmacological effect [81,96].

Experimental evidence further supports these observations. Abbas et al. evaluated sequential *P. harmala* extracts using complementary in vitro and in vivo inflammatory models and reported that the methanolic extract exhibited the strongest anti-inflammatory activity. In vitro, the crude methanolic extract inhibited bovine serum albumin denaturation by 63.0%, whereas bioassay-guided fractionation further increased the inhibitory activity to 71.7% for Fraction B and 72.9% for the RP-HPLC subfraction PHMF2 at 400 μg/mL. Similarly, PHMF2 produced the highest inhibition of heat-induced hemolysis (62.7%) and egg albumin denaturation (68.1%), indicating improved activity following fractionation [95].

The anti-inflammatory activity was further confirmed in vivo using carrageenan-induced paw edema. Oral administration of the methanolic extract at 200 mg/kg reduced paw edema by 75.14% after 3 h, approaching the effect of the reference drug indomethacin (86.3%). Likewise, the same extract reduced formaldehyde-induced paw edema by 76.32% after 24 h compared with 89.3% for indomethacin, whereas dichloromethane and hydroalcoholic extracts displayed substantially lower efficacy. These findings indicate that methanol efficiently extracts the compounds responsible for anti-inflammatory activity, which were subsequently identified as quinic acid, peganine, harmol, harmaline, and harmine following bioassay-guided purification [95].

The reported anti-inflammatory effects are likely attributable to the combined action of β-carboline alkaloids and phenolic compounds, which regulate multiple inflammatory pathways while simultaneously reducing oxidative stress. Because oxidative stress and inflammation are closely interconnected, the antioxidant properties of *P. harmala* may contribute indirectly to the attenuation of inflammatory responses. Nevertheless, the available evidence remains dominated by animal models and protein denaturation assays, which cannot fully reproduce the complexity of human inflammatory diseases. Furthermore, differences in extraction procedures, phytochemical composition, experimental models, and outcome measures limit direct comparisons among studies.

Future investigations should therefore combine standardized phytochemical characterization with mechanistic studies targeting NF-κB, COX-2, iNOS, and pro-inflammatory cytokines, followed by well-designed preclinical and clinical studies to validate the therapeutic potential of *P. harmala* in inflammatory disorders.

### 7.5. Antidiabetic Activity

*Peganum harmala* has emerged as a promising source of natural antidiabetic agents owing to its rich phytochemical composition and its ability to inhibit key carbohydrate-hydrolyzing enzymes involved in postprandial glucose regulation. Although several studies have highlighted the antidiabetic potential of *P. harmala* through enzyme inhibition and antioxidant mechanisms [1,2,3,4], quantitative evidence remains relatively scarce, with only a few investigations reporting standardized inhibitory activities. Ethanol extracts of *P. harmala* have demonstrated significant inhibitory activity against α-amylase and α-glucosidase, with inhibitory capacities of 2.76 mmol acarbose equivalents (ACAE)/g and 1.20 mmol ACAE/g, respectively. These findings suggest that the extract may effectively reduce carbohydrate digestion and glucose absorption, thereby contributing to improved glycemic control. In the same study, the extract also exhibited strong inhibitory effects against acetylcholinesterase (2.99 mg galantamine equivalents (GALAE)/g) and butyrylcholinesterase (4.14 mg GALAE/g), whereas only moderate inhibition of tyrosinase was observed (35.8 mg kojic acid equivalents (KAE)/g) [4]. The extract was characterized by the presence of several bioactive phytochemicals, including acacetin, rutin, apigenin, chrysin, quinic acid, gentisic acid, and *p*-coumaric acid, which are recognized for their antioxidant and enzyme-inhibitory properties. Molecular docking further supported these biological activities, with acacetin exhibiting strong binding affinities toward α-glucosidase and α-amylase, yielding docking scores of −8.8 and −7.8 kcal/mol, respectively, suggesting a favorable interaction with the catalytic sites of these enzymes. These results indicate that *P. harmala* may exert its antidiabetic activity through a dual mechanism involving both enzyme inhibition and antioxidant protection [4].

Despite these encouraging findings, current evidence remains limited to in vitro enzyme inhibition assays and computational analyses. Moreover, the antidiabetic efficacy of *P. harmala* may vary depending on extraction solvent, phytochemical composition, and geographical origin of the plant [88,97]. Future studies should therefore validate these observations in in vivo diabetic models and clinical trials, while identifying the compounds primarily responsible for glucose-lowering activity and elucidating their molecular mechanisms.

### 7.6. Anticancer Activity

*Peganum harmala* has attracted considerable attention as a source of potential anticancer agents owing to its high content of β-carboline alkaloids, particularly harmine, harmaline, harmol, and harmane, which exhibit multiple mechanisms of action against tumor cells [98,99,100,101]. These compounds have demonstrated antiproliferative, pro-apoptotic, anti-migratory, and anti-invasive activities across a wide range of cancer models, highlighting the multitarget pharmacological profile of the species [45,102].

Recent evidence further supports the anticancer potential of *P. harmala*. A comprehensive phytochemical and biological evaluation showed that the roots contained substantially higher concentrations of β-carboline alkaloids than the seeds, with harmine (151.90 ± 0.27 mg/g) and harmol (70.51 ± 0.11 mg/g) representing the predominant constituents, whereas seeds contained considerably lower levels of harmine (35.09 ± 1.41 mg/g). This phytochemical enrichment was associated with potent cytotoxic activity against several human cancer cell lines, including SiHa cervical cancer (IC_50_ = 6.05 μM), A2780 ovarian cancer (6.54 μM), MCF-7 breast cancer (8.06 μM), C33A cervical cancer (12.27 μM), UPCI-SCC-154 head and neck carcinoma (13.30 μM), HeLa cervical cancer (16.56 μM), MDA-MB-231 breast cancer (18.77 μM), and UPCI-SCC-131 cells (27.85 μM). Importantly, the extracts exhibited substantially lower toxicity toward normal NIH/3T3 fibroblasts (IC_50_ > 30 μM), suggesting a degree of selectivity for malignant cells [84].

Mechanistic investigations revealed that the anticancer activity of *P. harmala* involves multiple complementary pathways. β-carboline alkaloids induced apoptosis through activation of caspase-dependent signaling, promoted G2/M cell-cycle arrest, stabilized microtubules by interacting with tubulin, and significantly reduced cancer cell migration and invasion. These findings indicate that *P. harmala* not only suppresses tumor cell proliferation but may also interfere with metastatic progression, thereby broadening its therapeutic potential [57,84]. Among the biological activities reported for *P. harmala*, the anticancer activity is supported by some of the most potent quantitative in vitro outcomes reported to date, with IC_50_ values below 10 μM against several human cancer cell lines. These findings suggest that the β-carboline alkaloids of *P. harmala* represent one of the most promising pharmacological applications of this medicinal species.

Despite these promising findings, the current evidence remains largely limited to in vitro studies and experimental models. Considerable variability in plant origin, extraction procedures, alkaloid composition, and experimental protocols complicates comparisons among studies and hinders the identification of standardized therapeutic preparations. Moreover, although β-carboline alkaloids exhibit promising anticancer activity, additional studies are required to establish standardized therapeutic preparations and to better characterize their safety profile before clinical application can be considered.

Future studies should therefore focus on standardized phytochemical characterization, detailed mechanistic investigations, pharmacokinetic and toxicological evaluations, and well-designed in vivo and clinical studies. Particular attention should also be given to the development of optimized formulations and combination therapies that may enhance the efficacy of *P. harmala* alkaloids while minimizing potential adverse effects.

Despite the promising in vitro cytotoxic activity reported for *P. harmala* extracts and isolated alkaloids, several important challenges remain before clinical translation can be considered. Although some studies have reported lower toxicity toward normal cells, systematic evaluation of selectivity across different normal human cell types remains limited, making it difficult to accurately define the therapeutic window of these compounds. Furthermore, information regarding pharmacokinetics, bioavailability, metabolism, and tissue distribution is still scarce. Consequently, robust in vivo studies, pharmacokinetic investigations, and well-designed preclinical evaluations are required before the anticancer potential of *P. harmala* can be translated into clinically relevant therapeutic applications.

### 7.7. Mechanisms of Action

**Figure 3:**
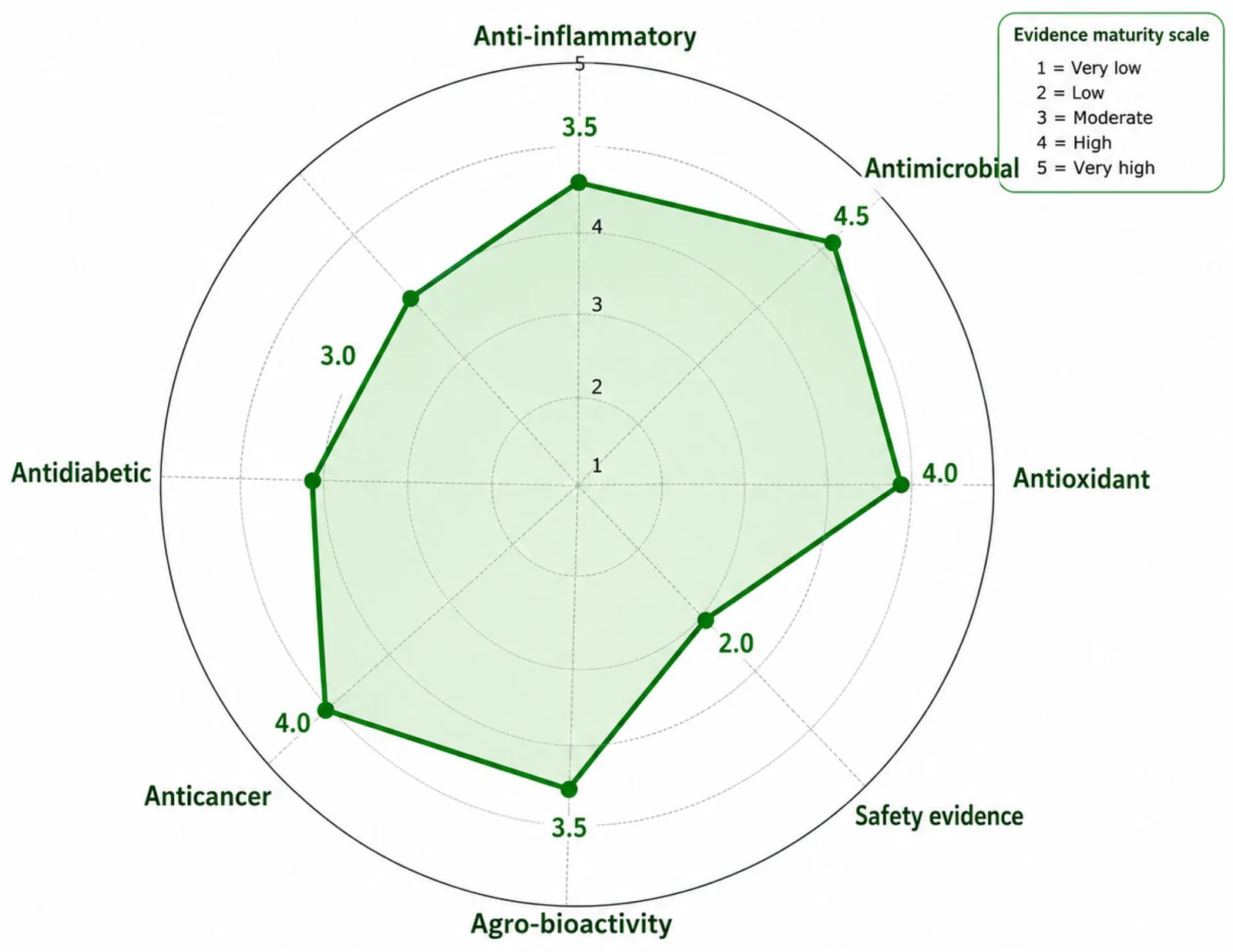
Relative maturity of biological evidence supporting the reported activities of *Peganum harmala* L.

Mechanistic interpretation should distinguish four levels: direct chemical reactivity, target-level interaction, cellular pathway modulation, and organism-level outcome. In *P. harmala*, β-carbolines may contribute through enzyme inhibition and signaling effects, phenolics through redox and inflammatory modulation, and volatile constituents through membrane disruption and vapor-phase interactions [46,47]. Synergy is plausible but often remains insufficiently demonstrated; therefore, claims of synergistic activity require fraction recombination, isobologram or checkerboard analyses, and quantitative characterization of each fraction [89]. The current literature is heterogeneous in terms of methodological quality and translational readiness across biological activity domains [36,96]. As summarized in Figure 3, antimicrobial and antioxidant activities are supported by the strongest body of evidence, whereas antidiabetic activity and, particularly, safety evaluation remain comparatively underexplored, highlighting important priorities for future research.

While Figure 3 provides an overview of the current level of evidence supporting the biological activities of *P. harmala*, a more detailed comparison is required to relate these activities to their major phytochemical contributors and the corresponding experimental validation strategies. The principal biological activities, together with their putative phytochemical contributors, recommended validation approaches, and representative references, are summarized in Table 6.

## 8. Toxicological Aspects and Safety Considerations

### 8.1. Toxicity of β-Carboline Alkaloids

The toxicity of *Peganum harmala* L. is primarily attributed to its β-carboline alkaloids, particularly harmine and harmaline, which are concentrated in the seeds and act as reversible monoamine oxidase A (MAO-A) inhibitors [1,2]. Besides their therapeutic potential, these alkaloids can induce significant neuropharmacological effects when consumed at excessive doses due to increased monoaminergic neurotransmission and interactions with multiple molecular targets, including serotonergic, dopaminergic, and imidazoline receptors [36,108].

Among the major alkaloids, harmaline is considered more toxic than harmine and has been reported to constitute approximately 3% of the seed composition [44,108]. Experimental studies have shown that harmaline may induce tremor, convulsions, respiratory depression, hypothermia, visual disturbances, delirium, impaired coordination, and central nervous system depression [108]. In contrast, harmine exhibits comparatively lower acute neurotoxicity but shares similar pharmacodynamic mechanisms [1,44]. These toxic manifestations are mainly associated with reversible MAO inhibition together with interactions with serotonin, dopamine, and benzodiazepine receptors [36,46].

Although purified β-carboline alkaloids possess greater neurotoxic potential because of their higher concentrations of active constituents, crude plant extracts generally exhibit lower acute toxicity [2]. For example, an acute toxicity study demonstrated that the methanolic extract of *P. harmala* showed no mortality at an oral dose of 3000 mg/kg in rodents, indicating an LD_50_ greater than 3000 mg/kg [95]. Likewise, repeated oral administration of the methanolic extract at doses of 500 and 1000 mg/kg for 28 days produced no significant alterations in body weight, organ weight, hematological parameters, or biochemical markers [95]. The oil extract also exhibited no observable toxicity at doses up to 320 mg/kg in experimental animals (Table 7) [95].

Despite these encouraging findings for crude extracts, standardized NOAEL and LOAEL values for *P. harmala* preparations or purified β-carboline alkaloids have not yet been established in the available literature [2,109]. Although the available evidence suggests toxicological differences among crude extracts, essential oils, and purified alkaloids, direct head-to-head experimental comparisons remain scarce [108], limiting accurate assessment of their relative toxicological profiles. Future studies following internationally accepted toxicological guidelines are therefore required to establish dose–response relationships and define safety thresholds for different *P. harmala* preparations [98].

### 8.2. Acute Toxicity and Clinical Manifestations

Human intoxication with *Peganum harmala* L. is relatively uncommon but has been repeatedly reported following the ingestion of seeds or concentrated preparations for traditional medicinal purposes [1,110]. Clinical manifestations are primarily dose-dependent and are largely attributed to the high content of β-carboline alkaloids, particularly harmaline and harmine, which exert potent reversible MAO-A inhibitory activity [108] and affect multiple neurotransmitter systems [2,35].

The nervous system is the principal target of acute toxicity [110]. Reported neurological manifestations include dizziness, confusion, agitation, tremor, ataxia, dysarthria, impaired coordination, hallucinations, decreased consciousness, and, in severe cases, convulsions or coma [110]. These effects are consistent with excessive serotonergic and dopaminergic stimulation together with direct actions of β-carboline alkaloids on central nervous system receptors [46,108]. Gastrointestinal symptoms, including nausea, vomiting, and abdominal discomfort, generally represent the earliest clinical manifestations after ingestion [110].

Cardiovascular disturbances have also been described, although they are generally reversible [110]. Hypotension is frequently observed following overdose because of the vasorelaxant properties of β-carbolines [1,108], whereas hypertension may occur when *P. harmala* is co-administered with tyramine-rich foods or drugs owing to MAO inhibition [35,100]. Respiratory depression and hypothermia have also been reported in severe poisoning episodes [110].

A representative clinical case involved a 45-year-old woman who ingested approximately 50 g of *P. harmala* seeds as a traditional remedy [110]. Within a few hours, she developed nausea, vomiting, dizziness, tremor, ataxia, confusion, and postural hypotension (90/60 mmHg) [110]. Laboratory investigations remained within normal limits, and the patient recovered completely after supportive treatment with intravenous fluids and observation, being discharged 18 h after admission [110]. Similar clinical manifestations have been consistently reported in previous intoxication cases, suggesting that most poisonings are reversible when appropriate medical care is promptly provided [110].

Overall, current clinical evidence indicates that the acute toxicity of *P. harmala* primarily affects the central nervous, gastrointestinal, and cardiovascular systems [1,110]. Although the prognosis is generally favorable with supportive management, severe poisoning may occur after ingestion of large quantities or when β-carboline alkaloids interact with medications or dietary amines [35,100]. These observations emphasize the importance of cautious therapeutic use and appropriate medical supervision.

### 8.3. Chronic Toxicity, Herb–Drug Interactions, and Safety Considerations

Although acute poisoning has been more extensively documented than chronic exposure, repeated consumption of *Peganum harmala* preparations may present additional toxicological concerns because of the cumulative pharmacological effects of β-carboline alkaloids [2,108]. Owing to their reversible inhibition of monoamine oxidase A (MAO-A), harmine and harmaline may interfere with the metabolism of endogenous monoamines as well as numerous therapeutic agents [36,46]. Consequently, prolonged or uncontrolled consumption of *P. harmala* preparations should be approached with caution, particularly in individuals receiving long-term pharmacological treatments [109].

The most clinically relevant safety concern is the potential for herb–drug interactions. β-carboline alkaloids may potentiate the pharmacological activity of antidepressants, selective serotonin reuptake inhibitors (SSRIs), serotonin–norepinephrine reuptake inhibitors (SNRIs), monoamine oxidase inhibitors, sympathomimetic drugs, and other serotonergic medications, thereby increasing the risk of serotonin syndrome or hypertensive crises [35,100,108]. Similar interactions may occur following the consumption of tyramine-rich foods because MAO inhibition reduces tyramine metabolism, resulting in excessive catecholamine release and elevated blood pressure [35,100].

In addition to pharmacodynamic interactions, β-carbolines may influence drug pharmacokinetics by modulating cytochrome P450 enzymes and membrane transporters involved in xenobiotic metabolism [100]. Although current evidence remains limited, these interactions may alter the absorption, metabolism, or elimination of co-administered drugs, thereby affecting their efficacy and safety [100,109]. Further clinical pharmacokinetic investigations are therefore required to characterize these interactions under therapeutic conditions.

Current evidence also suggests that the safety profile of *P. harmala* largely depends on the type of preparation, dosage, duration of exposure, and alkaloid concentration [2,108]. Standardized extracts with controlled phytochemical composition are expected to provide greater safety than crude homemade preparations, in which alkaloid content may vary considerably [101,109]. Consequently, rigorous quality control, dose standardization, contaminant monitoring, and comprehensive toxicological evaluation should be considered essential prerequisites before the widespread medicinal application of *P. harmala* preparations [98,101].

### 8.4. Future Perspectives and Research Needs

Despite the growing body of evidence regarding the pharmacological potential of *Peganum harmala*, several toxicological aspects remain insufficiently characterized. Most available studies have focused on acute toxicity or short-term experimental models, whereas comprehensive long-term investigations addressing chronic exposure, reproductive toxicity, developmental toxicity, genotoxicity, carcinogenicity, and immunotoxicity remain scarce [2,108,109]. Establishing these toxicological endpoints is essential for accurately defining the safety profile of *P. harmala* and its bioactive constituents.

Another major limitation is the considerable variability in alkaloid content among different plant organs, geographical origins, harvesting periods, and extraction methods [1,22,108]. Such variability complicates comparisons among studies and hinders the establishment of standardized therapeutic dosages. Future investigations should therefore emphasize phytochemical standardization and rigorous quality control to improve the reproducibility and safety of *P. harmala*-based products [98,101].

In addition, well-designed pharmacokinetic and clinical studies are needed to better understand the absorption, distribution, metabolism, excretion, and potential herb–drug interactions of β-carboline alkaloids in humans [54,100]. The establishment of standardized NOAEL, LOAEL, and acceptable daily intake (ADI) values according to internationally recognized toxicological guidelines would further facilitate risk assessment and support future regulatory approval [98,109].

Finally, advances in analytical chemistry, metabolomics, and systems pharmacology offer promising opportunities to improve the characterization of *P. harmala* preparations and identify safer therapeutic formulations with optimized efficacy-to-toxicity ratios [51,69,111]. Integrating these multidisciplinary approaches with robust preclinical and clinical investigations will be essential for translating the therapeutic potential of *P. harmala* into evidence-based pharmaceutical and nutraceutical applications while ensuring patient safety [101,108,112].

**Figure 4:**
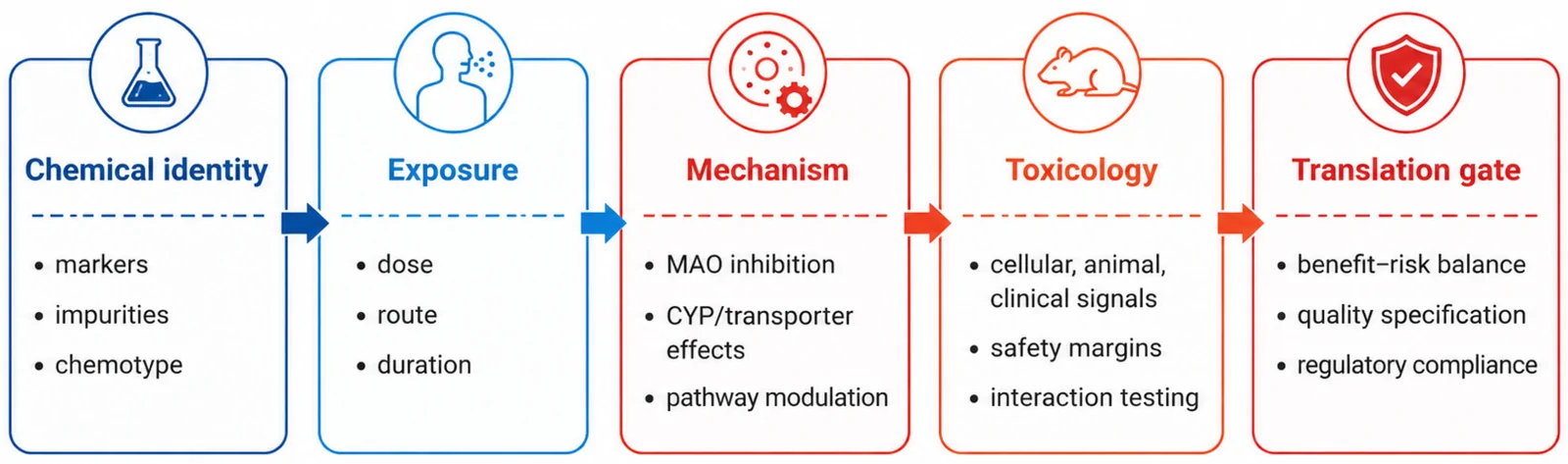
Safety and translation gate for *Peganum harmala* products and lead compounds, emphasizing that chemical identity, exposure, mechanism and toxicology must be assessed before application claims.

While Figure 4 provides an overview of the safety-first framework for *P. harmala*, a more detailed evaluation of the principal toxicological dimensions is required to support translational development. The major safety concerns, their principal risk drivers, and the minimum evidence recommended for regulatory-grade evaluation are summarized in Table 8.

## 9. Industrial and Pharmaceutical Applications

### 9.1. Pharmaceutical Applications

Pharmaceutical interest in *P. harmala* should be framed as lead discovery and mechanism exploration rather than direct therapeutic endorsement [47,48,104]. Harmine, harmaline and related beta-carbolines provide scaffolds for medicinal chemistry, target validation and structure–activity relationship studies [113]. Standardized extracts may also be useful as research tools, but pharmaceutical development requires purification, selectivity, pharmacokinetic optimization, formulation, toxicology and clinical testing [112,114].

Essential oil and volatile fractions may contribute to antimicrobial, anti-inflammatory or delivery-related applications, but pharmaceutical use requires tight quality control [59]. Batch variability, oxidation, photodegradation and volatility can compromise reproducibility [113,115]. Encapsulation into lipid carriers, hydrogels or nanoemulsions can improve stability and delivery, yet it also introduces new safety and characterization requirements. Formulation cannot compensate for an unstandardized extract [116,117,118].

### 9.2. Nutraceutical Applications

Nutraceutical development is the most problematic application category for *P. harmala* because oral exposure to beta-carboline-rich preparations may present safety and interaction concerns [4,35]. Although antioxidant or enzyme-inhibitory activities can appear attractive, nutraceutical claims require compositional standardization, low-toxicity fractions, exposure limits and clinical substantiation [98]. At present, *P. harmala* is better approached as a source of characterized molecules than as a generalized dietary supplement [101].

### 9.3. Cosmetic Applications

Cosmetic applications may be plausible for low-exposure, well-characterized, non-irritant fractions with antioxidant, antimicrobial or preservative-supporting properties [115,119]. However, cosmetic translation must consider odor, color, skin irritation, phototoxicity, alkaloid content and regulatory ingredient restrictions [120,121]. Plant-based cosmetic oils and natural product formulations demonstrate that botanical origin alone is insufficient; performance, safety and stability must be quantified [122].

### 9.4. Food Preservation Potential

Essential oils are widely explored as natural antimicrobial agents in food preservation, active packaging and edible coatings. *P. harmala* volatile fractions could be investigated in this context, but alkaloid contamination and sensory intensity must be tightly controlled. For food-related applications, vapor-phase antimicrobial activity, migration into food matrices, toxicological exposure and regulatory acceptance are decisive. Active packaging may offer safer exposure control than direct ingestion [91,92,93,123,124].

### 9.5. Agricultural Applications

Agricultural applications include botanical insecticides, antifungal treatments, weed management and allelopathy-inspired bioprotection. *P. harmala* contains phytotoxic and insecticidal chemical features, but field relevance requires non-target organism testing, environmental persistence studies, formulation stability and dose optimization. A sustainable agricultural product must not simply move toxicity from synthetic chemicals to unstandardized botanicals; it must provide predictable efficacy with controlled ecological risk [23,24,105,106,107].

The successful translation of *P. harmala* from experimental studies to real-world applications depends on balancing biological efficacy with safety, regulatory compliance, formulation stability, and industrial scalability. Although numerous bioactivities have been reported, each application area faces distinct scientific and technological challenges. Table 9 summarizes the principal opportunities, major barriers, and recommended development pathways for the responsible utilization of *P. harmala*-derived products.

## 10. Quality-by-Design (QbD) Framework for *Peganum harmala* L.

The increasing interest in *Peganum harmala* for pharmaceutical, cosmetic, agricultural, and food-related applications highlights the need for a systematic strategy capable of ensuring product quality, reproducibility, and safety throughout the entire development process. Although numerous studies have investigated its phytochemical composition, extraction technologies, biological activities, and toxicological properties, these aspects are generally addressed independently, limiting the translation of experimental findings into standardized and reproducible products. A Quality-by-Design (QbD) approach provides a comprehensive framework that integrates these complementary research areas by considering quality as a property that must be built into the product from the earliest stages of development rather than verified only through final quality control.

Within this framework, the quality of *P. harmala*-derived products is determined by the interaction between the characteristics of the botanical raw material, extraction methodology, analytical characterization, biological performance, and toxicological profile. Botanical authentication, organ-specific phytochemical variability, geographical origin, harvesting conditions, extraction parameters, and analytical quality control collectively influence the final chemical composition and, consequently, the biological efficacy and safety of the resulting extracts or essential oils. Therefore, these variables should not be considered independently but as interconnected components of an integrated quality system.

The evidence synthesized throughout this review demonstrates that variations in plant organ, chemotype, environmental conditions, extraction technology, and analytical methodology can substantially modify the concentrations of β-carboline alkaloids, volatile constituents, phenolic compounds, and other bioactive metabolites. Such variability directly affects biological activity, toxicological behavior, and the reproducibility of experimental results. Consequently, adopting a QbD framework provides a rational basis for identifying the variables that most strongly influence product quality, prioritizing critical process controls, and facilitating the development of standardized *P. harmala*-based products suitable for future pharmaceutical, cosmetic, agricultural, and industrial applications.

### 10.1. Rationale for a Quality-by-Design Approach

The development of botanical products from *Peganum harmala* presents unique challenges because the biological activity of the plant depends on a highly variable phytochemical profile influenced by both natural and technological factors [2]. Unlike single-compound pharmaceutical products, botanical extracts contain multiple classes of bioactive constituents whose relative abundance may vary according to plant organ, geographical origin, harvesting conditions, post-harvest processing, extraction technology, and analytical methodology [4,5]. As discussed throughout this review, these sources of variability complicate direct comparisons among published studies and often limit the reproducibility of reported biological activities [22,33,56].

A Quality-by-Design approach addresses these limitations by systematically identifying the factors that influence product quality before large-scale development [5,6,7,8,9]. Rather than relying exclusively on end-product testing, QbD emphasizes the understanding and control of both material-related and process-related variables throughout the production chain [15,16,17,18,19,20]. For *P. harmala*, this strategy facilitates the establishment of scientifically justified relationships between raw material characteristics, extraction conditions, phytochemical composition, biological performance, and toxicological safety [70,79]. Such an integrated framework is particularly important for β-carboline-rich medicinal plants, where slight variations in chemical composition may significantly influence both pharmacological efficacy and toxicological risk [35,36,53,54,96,98,99,100,101,109,110].

The following sections apply the principles of Quality-by-Design to *P. harmala* by identifying the Critical Material Attributes (CMAs), Critical Process Parameters (CPPs), and Critical Quality Attributes (CQAs) that should be considered during the development of standardized botanical products. This framework also provides the basis for risk assessment and future Design of Experiments (DoE)-guided optimization strategies aimed at improving product consistency, safety, and industrial scalability.

### 10.2. Critical Material Attributes (CMAs)

Critical Material Attributes (CMAs) refer to the inherent characteristics of the botanical raw material that significantly influence the quality, consistency, and safety of the final product [5,6]. In the context of *Peganum harmala*, these attributes encompass all plant-related factors capable of affecting the phytochemical composition prior to extraction and, consequently, the biological performance of the resulting extracts or essential oils [21,22]. Since *P. harmala* exhibits considerable chemical variability, the identification and control of CMAs represent a fundamental step in the implementation of a Quality-by-Design strategy [33,56].

Among the identified CMAs, the plant organ constitutes one of the most influential factors. As discussed in Section 4, seeds, roots, stems, leaves, and flowers differ markedly in their concentrations of β-carboline alkaloids, phenolic compounds, and volatile constituents [33]. Seeds are generally recognized as the richest source of harmine and harmaline, whereas aerial parts and roots exhibit distinct phytochemical profiles. Such organ-specific variability directly influences extraction yield, chemical composition, biological activity, and toxicological properties, making accurate botanical selection essential for product standardization [44].

Geographical origin and environmental conditions also represent critical sources of variability [21,22]. Differences in climate, soil composition, altitude, and local ecological conditions may substantially modify the accumulation of secondary metabolites, leading to significant variations in alkaloid content, essential oil composition, and antioxidant constituents among populations originating from different regions. Consequently, geographical traceability should be considered an important quality attribute during the selection of botanical raw materials [38,61].

Additional CMAs include harvest maturity, post-harvest drying, and storage conditions. Harvest stage influences metabolite accumulation, while inappropriate drying or prolonged storage may promote the degradation of thermolabile or volatile constituents, thereby affecting both phytochemical composition and biological activity [15,16]. Standardization of these pre-processing conditions is therefore essential to minimize batch-to-batch variability and preserve the integrity of bioactive compounds [21,22].

From a QbD perspective, comprehensive characterization of these Critical Material Attributes provides the scientific foundation for selecting appropriate extraction conditions and establishing reproducible quality standards for *P. harmala*-derived products. Their systematic control contributes to improved product consistency, facilitates subsequent process optimization, and ultimately supports the development of safer and more reliable botanical preparations.

### 10.3. Critical Process Parameters (CPPs)

Critical Process Parameters (CPPs) are the operational variables that directly influence the efficiency of extraction, the recovery of bioactive compounds, and the overall quality of the final botanical product [17,18,19,20]. In a Quality-by-Design framework, these parameters should be systematically identified, controlled, and optimized to ensure the reproducibility of *Peganum harmala*-derived extracts and essential oils [59,70]. Since the phytochemical composition of *P. harmala* is highly sensitive to extraction conditions, process optimization represents a key determinant of both biological performance and product safety [71,79].

Among the principal CPPs, the extraction technique constitutes one of the most influential variables [5,6,7,8,9]. Conventional methods such as hydrodistillation, steam distillation, and solvent extraction differ considerably from emerging green technologies, including ultrasound-assisted extraction (UAE), microwave-assisted extraction (MAE), and supercritical carbon dioxide (SC-CO_2_) extraction, in terms of extraction efficiency, selectivity, and preservation of thermolabile constituents [17,18,19,20]. As discussed in Section 5, each extraction technique produces extracts with distinct phytochemical profiles, thereby influencing their subsequent biological activities [59,70].

Process variables such as extraction temperature, pressure, extraction time, solvent composition, solvent-to-material ratio, and particle size further affect the recovery of β-carboline alkaloids, phenolic compounds, and volatile constituents [15,16]. Inappropriate extraction conditions may promote the degradation of sensitive metabolites, reduce extraction efficiency, or alter the relative abundance of key phytochemical markers, ultimately compromising batch-to-batch reproducibility and biological consistency [17,18,19,20]. Consequently, these variables should be carefully optimized according to the intended application of the final product [70,71,79].

Within a QbD strategy, CPPs should not be optimized independently but rather in conjunction with the Critical Material Attributes of the botanical raw material. The interaction between raw material variability and extraction conditions ultimately determines the phytochemical composition, biological activity, and safety profile of *P. harmala* preparations. Therefore, systematic control of CPPs represents an essential step toward establishing robust, reproducible, and scalable extraction processes suitable for pharmaceutical, cosmetic, food, and agricultural applications.

### 10.4. Critical Quality Attributes (CQAs)

Critical Quality Attributes (CQAs) are the measurable physicochemical and biological characteristics that determine the identity, consistency, efficacy, and safety of the final botanical product [33,37,39,40,41,42,43,49]. Within a Quality-by-Design framework, CQAs represent the quality targets that should be achieved through the appropriate control of both Critical Material Attributes (CMAs) and Critical Process Parameters (CPPs) [35,36,53,54,96]. For *Peganum harmala*, the definition of relevant CQAs is particularly important because the plant contains pharmacologically active β-carboline alkaloids whose concentrations may vary considerably depending on both botanical and processing factors [98,99,100,101,109,110].

The concentrations of harmine and harmaline constitute the primary CQAs for *P. harmala*-derived products because these alkaloids are largely responsible for the plant’s reported pharmacological activities while also contributing to its toxicological profile [42]. In addition to their individual concentrations, the harmine-to-harmaline ratio may serve as a valuable quality indicator reflecting phytochemical consistency between production batches [44,45]. Likewise, the qualitative and quantitative composition of essential oils, together with the abundance of major volatile constituents, represents another important quality attribute for products intended for aromatic, cosmetic, or pharmaceutical applications [35,36,53,54,96,98,99,109,110].

Other relevant CQAs include the overall phytochemical profile, extract purity, and the concentration of potentially toxic alkaloids [35,36,53,54,96]. Since excessive levels of β-carboline alkaloids may increase the risk of adverse effects, maintaining their concentrations within reproducible and scientifically justified limits is essential for ensuring product safety [98,109,110]. The preservation of characteristic phytochemical fingerprints also contributes to batch-to-batch reproducibility and facilitates analytical quality control [99,100,101].

Finally, biological performance should be considered an integral component of product quality [55,56,73,102,103]. Reproducible antioxidant, antimicrobial, anti-inflammatory, and other pharmacological activities should be supported by consistent phytochemical composition rather than evaluated independently [46,47,48,104]. Consequently, the systematic control of CQAs provides the foundation for producing standardized *P. harmala* extracts with predictable efficacy, reproducible quality, and improved safety, thereby facilitating their future pharmaceutical and industrial development [88,97,126,127].

### 10.5. Risk Assessment and Design Space

Within a Quality-by-Design framework, risk assessment provides a systematic approach for identifying the factors that are most likely to influence the Critical Quality Attributes (CQAs) of *Peganum harmala*-derived products [128,129]. Rather than considering all variables as equally important, risk assessment enables the prioritization of material- and process-related factors according to their potential impact on phytochemical composition, biological activity, and safety [5,6,7,8,9]. This preliminary evaluation provides a rational basis for process optimization and the establishment of robust quality control strategies [71,72,74,79,80,91,130].

Based on the evidence summarized throughout this review, several variables can be identified as presenting different levels of risk to product quality (Table 10). Plant organ, chemotype, and extraction solvent are considered high-risk factors because they directly determine the qualitative and quantitative composition of β-carboline alkaloids, essential oil constituents, and other bioactive metabolites. Extraction temperature and post-harvest drying conditions represent medium-risk factors, as inappropriate processing may promote degradation or loss of thermolabile compounds. Storage conditions are generally associated with a lower level of risk, although prolonged or unsuitable storage may still affect the stability of volatile constituents and overall phytochemical integrity.

**Figure 5:**
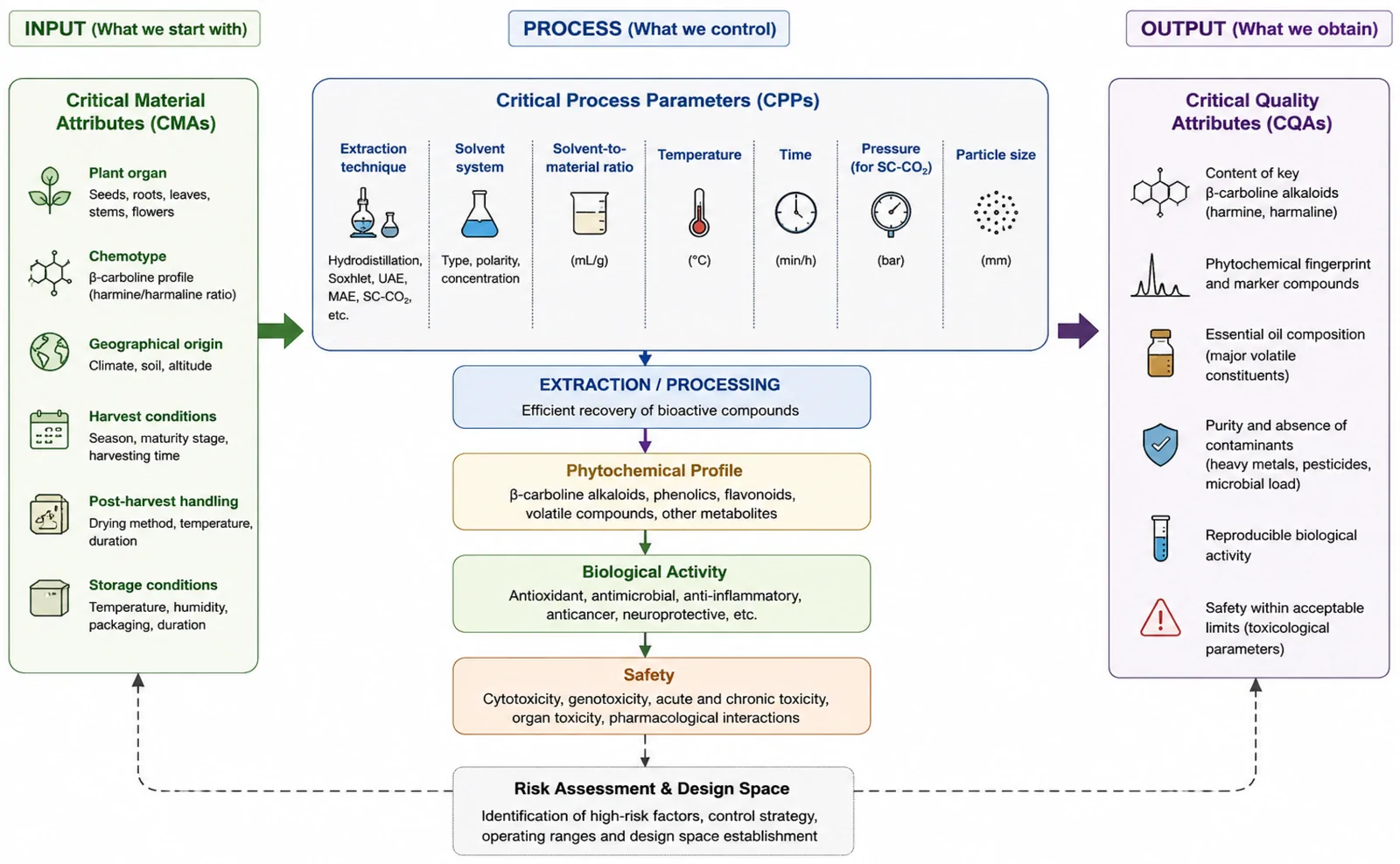
Conceptual Design Space for *Peganum harmala*-derived products. Solid arrows indicate the progression from material and process variables toward critical quality attributes, while dashed arrows represent feedback from risk assessment and design-space evaluation.

The identification of these critical factors contributes to the definition of a preliminary design space in which the interactions between botanical raw materials, extraction parameters, chemical composition, biological activity, and product safety can be understood and controlled. Although the establishment of a validated design space requires experimental optimization, the conceptual framework proposed in this review illustrates how systematic control of Critical Material Attributes (CMAs) and Critical Process Parameters (CPPs) ultimately determines the Critical Quality Attributes (CQAs) of standardized *P. harmala* products, thereby supporting their reproducibility, quality, and safety. As illustrated in Figure 5, the quality of *P. harmala* products results from the interaction between the characteristics of the botanical raw material, the extraction process, and the resulting phytochemical profile. This conceptual design space highlights how the systematic control of CMAs and CPPs contributes to achieving the desired CQAs, thereby supporting product quality, reproducibility, and safety.

## 11. Research Gaps and Future Perspectives

### 11.1. Need for Standardized Extraction Protocols

Based on a critical analysis of the available literature, the following recommendations are proposed by the authors to improve the standardization and reproducibility of future *Peganum harmala* studies [11]. These recommendations should be considered as evidence-informed perspectives rather than established consensus guidelines [15,16].

Standardization is the first priority for the field. The review recommends that every future *P. harmala* extraction study report botanical voucher, organ, origin, harvest date, drying conditions, particle size, solvent or distillation parameters, extraction yield, storage conditions, and a complete analytical workflow. Without this minimum dataset, the literature will continue to accumulate isolated results that cannot be compared or translated.

### 11.2. Advanced Analytical Characterization

Advanced characterization should integrate GC-MS for volatiles, LC-MS/MS for alkaloids and phenolics, NMR for structural and quantitative confirmation, and molecular networking for unknown prioritization [58,65,66,67,68]. The goal is not to use sophisticated tools for their own sake, but to build chemical traceability from plant organ to extract to biological effect. Such traceability is the foundation of reproducible natural product science [51,62,111].

### 11.3. Green Extraction Technologies

Green extraction should be evaluated by multi-criteria metrics, not by method labels alone [17,18,19,20]. Energy use, solvent toxicity, extraction time, yield, marker recovery, chemical degradation, scalability and operator safety all matter. UAE, MAE and SC-CO_2_ should be compared directly with hydrodistillation and solvent extraction using matched biomass [70]. The best method will depend on whether the target is essential oil, alkaloid-rich fraction, phenolic fraction or formulated product [74,79].

### 11.4. Clinical Investigations

Clinical investigation is premature for most *P. harmala* preparations unless preceded by standardized composition, safety pharmacology, toxicology and exposure modeling [35]. Isolated compounds or carefully defined fractions may eventually be evaluated for specific indications, but crude preparations should not bypass the translational sequence [98,99]. For now, the most valuable near-term clinical contribution is not intervention testing but safety documentation, interaction assessment and exposure risk modeling [100,101].

### 11.5. Future Industrial Opportunities

**Figure 6:**
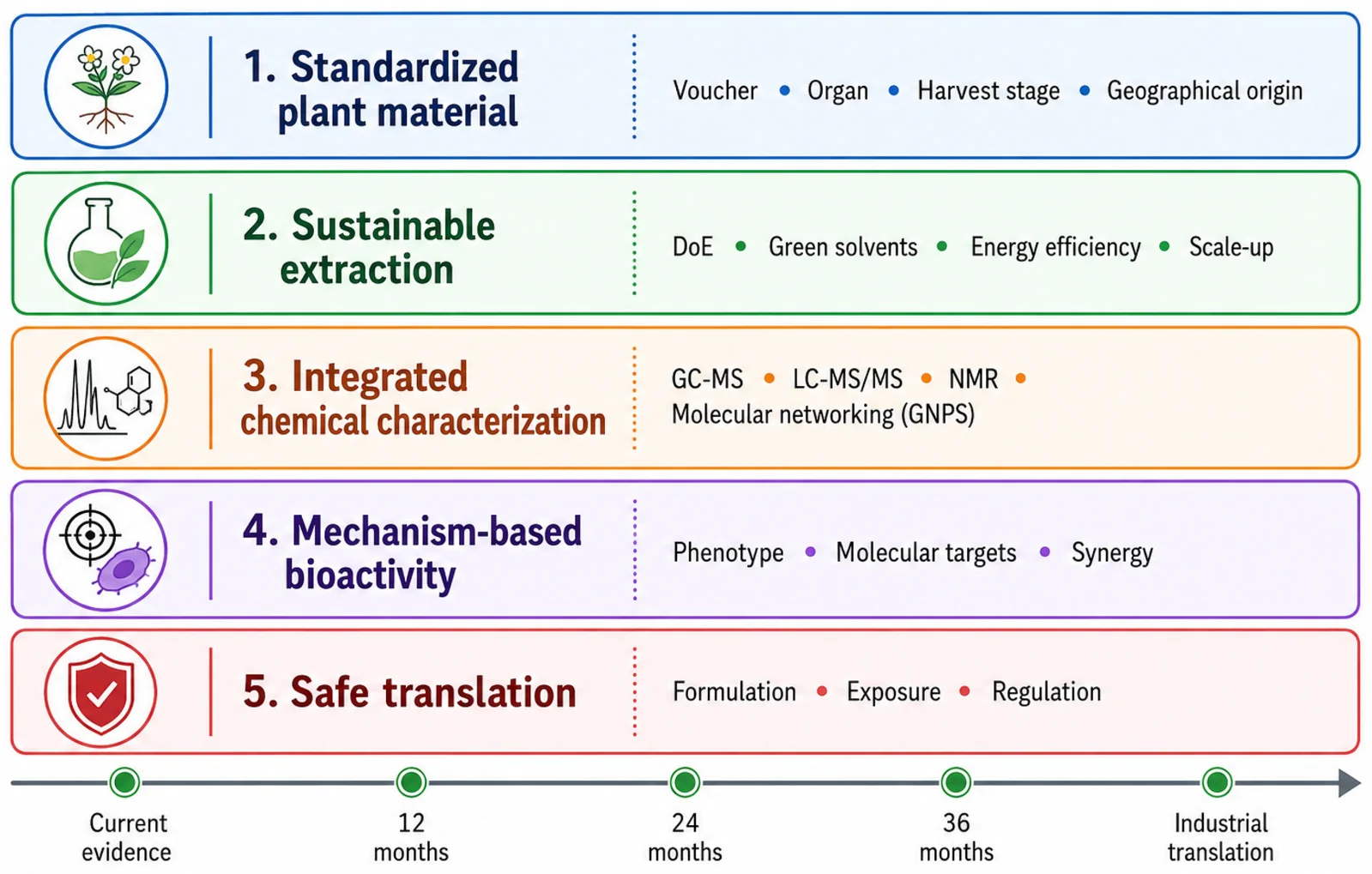
Future research roadmap for *Peganum harmala*, illustrating the key priorities required to achieve standardized biomass production, sustainable extraction, integrated chemical characterization, mechanism-based bioactivity evaluation and safe industrial translation [111,112,114,125].

The perspectives presented in this section reflect the authors’ interpretation of the current evidence and are intended as evidence-informed recommendations for future research and industrial translation rather than established regulatory requirements or consensus guidelines [113,115]. Future industrial opportunities are strongest where exposure can be controlled and chemical standardization is feasible, including analytical reference materials, lead compound libraries, topical formulations, agricultural bioprotectants, active packaging, and research-grade standardized extracts [93,116,117,118,123,124]. Long-term progress will depend on chemotype mapping, cultivation protocols, green extraction scale-up, formulation stability, and regulatory alignment [111,125]. As summarized in Figure 6, the future development of *P. harmala* requires an integrated strategy based on standardized plant material, sustainable extraction, advanced chemical characterization, mechanism-based bioactivity evaluation, and safe industrial translation. *P. harmala* has the potential to become a model species for the responsible development of potent ethnomedicinal plants, provided that safety, quality, and standardization are considered as design variables from the earliest stages of research and product development.

Table 11 summarizes the major research gaps, priority actions, and expected outcomes for advancing *P. harmala* from laboratory research toward safe and sustainable industrial applications.

## 12. Conclusions

*Peganum harmala* L. is a chemically distinctive medicinal and aromatic plant whose scientific value lies at the intersection of ethnopharmacology, β-carboline chemistry, volatile profiling, green extraction technologies, and safety-oriented product development. This review highlights that the chemical composition, biological activities, and translational potential of *P. harmala* cannot be interpreted independently of plant organ, extraction strategy, geographical origin, analytical characterization, and toxicological profile. Reliable future investigations should therefore integrate botanical authentication, standardized extraction protocols, comprehensive GC-MS and LC-MS/MS characterization, mechanism-based bioactivity evaluation, and rigorous dose–response and safety assessments.

The greatest opportunities for the development of *P. harmala* lie not in generalized therapeutic claims, but in evidence-based applications, including lead compound discovery, analytical reference materials, standardized research extracts, topical formulations, active packaging systems, and agricultural bioprotectants. Achieving these goals will require a quality-by-design approach in which chemical standardization, reproducibility, safety evaluation, and regulatory considerations are integrated from the earliest stages of research and product development. With such a multidisciplinary framework, *P. harmala* has the potential to serve as a valuable model for the responsible translation of bioactive medicinal plants into safe, standardized, and scientifically credible natural product platforms.

## Figures and Tables

**Table 1:** Eligibility criteria and evidence synthesis strategy adopted in the present review.

Criterion	Included Evidence	Excluded Evidence	Scientific Rationale
**Botanical authentication**	Studies providing authenticated *Peganum harmala* L. specimens with clearly identified plant organs and collection information.	Ambiguous botanical identity, unverified taxonomy, or unidentified plant material.	Accurate botanical authentication is essential for reproducibility and meaningful comparison among phytochemical studies.
**Phytochemical characterization**	Studies reporting comprehensive phytochemical profiling using GC-MS, LC-MS/MS, HPLC, NMR, molecular networking, or equivalent analytical techniques.	Biological studies lacking chemical characterization or extract profiling.	Reliable chemical characterization is fundamental for establishing composition–activity relationships.
**Essential oil characterization**	Studies reporting essential-oil yield, volatile composition, and major constituents using validated analytical methods.	Studies lacking essential oil composition or volatile profiling.	Standardized essential oil characterization enables meaningful comparison among different geographical origins and extraction methods.
**Extraction methodology**	Conventional and green extraction methods including hydrodistillation, steam distillation, solvent extraction, UAE, MAE, SC-CO_2_ and comparable technologies.	Studies lacking sufficient methodological details regarding extraction conditions.	Extraction methodology directly influences yield, selectivity, and phytochemical composition.
**Biological evidence**	Studies including appropriate controls, dose–response evaluation, statistical analysis, and interpretable biological endpoints.	Descriptive studies with inadequate controls, unclear experimental design, or unsupported biological claims.	High-quality experimental design is essential for reliable biological interpretation.
**Safety and toxicology**	Studies evaluating toxicity, exposure, pharmacological safety, dose–response relationships, or herb–drug interactions.	Unsupported therapeutic claims without toxicological evidence.	Safety assessment is indispensable for the responsible development of *P. harmala*-based products.
**Reporting quality**	Peer-reviewed studies providing sufficient methodological and analytical information for critical evaluation.	Publications with incomplete methodological reporting or insufficient experimental detail.	Comprehensive reporting improves transparency, reproducibility, and overall evidence quality.

**Table 2:** Recommended reporting variables for botanical, ecological, and ethnopharmacological studies of *Peganum harmala* L.

Variable	Scientific Rationale	Recommended Reporting Practice	Key References
**Taxonomy and voucher specimen**	Prevents misidentification and ensures botanical reproducibility.	Report scientific name, authority, family, voucher number, herbarium, and botanical authentication.	[1,2,3]
**Plant organ**	Seeds, leaves, roots, fruits, and aerial parts exhibit distinct phytochemical profiles.	Clearly specify the plant organ, developmental stage, and processing status.	[5,6,8,33]
**Geographical origin**	Climate, altitude, and soil conditions influence alkaloid accumulation and volatile composition.	Report collection site, GPS coordinates, altitude, climate, and soil characteristics.	[21,22,26]
**Harvest timing**	Phenological stage significantly affects essential oil yield and metabolite composition.	Specify harvest date, phenological stage, drying conditions, and storage period.	[15,16,38]
**Traditional preparation route**	Ethnopharmacological effects vary according to the route of administration and preparation method.	Distinguish fumigation, decoction, infusion, topical, and oral preparations.	[1,2,35,37]
**Ecological context**	Habitat characteristics may influence chemical defense mechanisms and secondary metabolism.	Describe habitat type, disturbance level, associated vegetation, and ecological conditions.	[23,24,25,30]

**Table 3:** Organ-specific phytochemical characteristics of *Peganum harmala* L. and recommended analytical approaches.

Plant Organ/Fraction	Major Phytochemical Characteristics	Recommended Analytical Approach	Interpretation and Recommendations
**Seeds**	Rich in **β-carboline alkaloids**, fixed oil, and volatile/semi-volatile constituents.	HPLC-DAD, HPLC-MS/MS or LC-MS/MS for alkaloids; GC-MS or HS-SPME-GC-MS for volatile profiling.	Seed chemistry should not be extrapolated to other plant organs because organ-specific phytochemical variation may be substantial [5,7,42].
**Roots**	Potential source of structurally diverse **β-carboline alkaloids** and bioactive secondary metabolites.	LC-MS/MS, HRMS, NMR, compound isolation, and bioactivity-guided fractionation.	Root harvesting may compromise plant sustainability, and root phytochemistry may differ considerably from that of seeds and aerial parts [33,39].
**Leaves**	Essential oil constituents, phenolic compounds, flavonoids, and high potential for aerial biomass valorization.	Hydrodistillation or HS-SPME followed by GC-MS for volatile analysis; LC-MS/MS for phenolics and flavonoids.	Leaf essential oils require independent chemical characterization and biological evaluation rather than inference from seed extracts [6,15,16].
**Fruits (Capsules)**	Developmental changes in alkaloid composition and potential quality markers associated with fruit maturation.	HRMS, LC-MS/MS, NMR, and targeted quantitative analysis.	Limited information is currently available; additional phytochemical studies are needed before biological extrapolation [33].
**Smoke/fumigation products**	Volatile constituents, combustion-derived products, and smoke condensates generated during traditional fumigation.	Smoke condensate characterization, headspace GC-MS, exposure assessment, and toxicological evaluation.	Traditional fumigation should be considered separately from oral or solvent extracts because exposure pathways and chemical composition differ substantially [35,37].

**Table 4:** Comparative overview of analytical platforms used for the phytochemical characterization of *Peganum harmala* L.

Analytical Platform	Primary Application	Major Strengths	Main Limitations
**GC-MS**	Profiling of volatile and semi-volatile constituents, particularly essential oils.	High sensitivity, extensive spectral libraries, retention-index comparison, and robust qualitative profiling.	Requires authentic standards for confirmation; co-eluting compounds may complicate identification [15,16].
**HS-SPME-GC-MS**	Headspace analysis of volatile compounds without hydrodistillation.	Solvent-free, highly sensitive for aroma profiling, minimal sample preparation.	Fiber selection, extraction equilibrium, and environmental conditions strongly influence reproducibility [5,8,69].
**HPLC-DAD**	Quantification of β-carboline alkaloids and targeted phytochemical analysis.	Simple, reproducible, cost-effective, and suitable for routine quality control.	Limited structural information for unknown compounds without complementary MS analysis [42].
**LC-MS/MS**	Targeted and untargeted analysis of alkaloids, phenolics, flavonoids, and other polar metabolites.	Excellent sensitivity, selectivity, structural information, and quantitative performance.	Matrix effects, ion suppression, and dependence on reference standards for absolute quantification [51,67].
**NMR/qNMR**	Structural elucidation, metabolite confirmation, purity assessment, and quantitative quality control.	Highly reproducible, non-destructive, and provides definitive structural information.	Lower sensitivity than mass spectrometry for trace constituents; requires relatively high sample amounts [62].
**Molecular networking (GNPS)**	Organization and visualization of chemical families; annotation of related metabolites.	Facilitates dereplication, discovery of unknown analogues, and prioritization of compounds for isolation.	Requires high-quality MS/MS data and expert interpretation; annotations remain putative without experimental confirmation [58,65,68].

**Table 5:** Comparative evaluation of conventional and emerging extraction technologies for *Peganum harmala* L. phytochemicals.

Extraction Technology	Major Strengths	Main Limitations	Recommended Applications
**Hydrodistillation (HD)**	Reference method for essential oil isolation; standardized procedure; facilitates comparison with previous studies.	Long extraction time, thermal degradation, and possible loss of highly volatile constituents.	Essential oil recovery, chemotaxonomic studies, and comparative investigations [8,9,59].
**Steam distillation (SD)**	Scalable process with reduced direct contact between plant material and boiling water; suitable for industrial production.	Thermal processing may underestimate polar or highly water-soluble constituents.	Pilot- and industrial-scale recovery of volatile compounds [70,71,72].
**Solvent extraction**	High recovery of β-carboline alkaloids, phenolics, and other semi-polar metabolites; broad phytochemical coverage.	Solvent residues, limited selectivity, and unsuitable for true essential oil characterization.	Bioactivity-guided fractionation, alkaloid isolation, and phytochemical profiling [42,55,73].
**Ultrasound-assisted extraction (UAE)**	Rapid extraction, reduced solvent consumption, improved mass transfer, and mild operating conditions.	Extraction efficiency depends on ultrasound power, extraction time, and temperature optimization.	Green extraction of alkaloids and phenolic compounds under optimized conditions [17,19,74].
**Microwave-assisted extraction (MAE)**	Short extraction time, improved energy efficiency, and enhanced extraction kinetics.	Local overheating may promote degradation of thermolabile metabolites if conditions are not carefully optimized.	Rapid laboratory-scale extraction and comparison with conventional techniques [20,80].
**Supercritical CO_2_ extraction (SC-CO_2_)**	Green solvent technology with tunable selectivity, solvent-free extracts, and excellent preservation of thermolabile compounds.	High equipment cost, optimization of pressure/temperature/co-solvent conditions, and limited efficiency for highly polar compounds without modifiers.	High-value extracts, industrial production, pharmaceutical and cosmetic applications, and sustainable process development [18,79].

**Table 6:** Comparative overview of the principal biological activities of *Peganum harmala* L., their major phytochemical contributors, recommended validation strategies, and representative references.

Biological Activity	Major Phytochemical Contributors	Recommended Validation Strategy	Representative References
**Antioxidant**	Phenolic compounds, flavonoids, and β-carboline alkaloids with redox-modulating properties.	Combine multiple in vitro antioxidant assays with cellular oxidative stress models and correlate activity with phytochemical composition.	[4,55,57,81]
**Antimicrobial**	Essential oil constituents, β-carboline alkaloids, and phenolic co-factors.	Determine MIC/MBC values using standardized broth microdilution methods, include solvent controls, reference antibiotics, cytotoxicity evaluation, and resistant strains when appropriate.	[57,73,84,89,90]
**Antifungal**	Volatile constituents, β-carboline alkaloids, and membrane-active phytochemicals.	Differentiate fungistatic from fungicidal effects, perform vapor-phase assays when relevant, and compare activity with conventional antifungal agents.	[89,92,93,94]
**Anti-inflammatory**	β-carboline alkaloids (harmine, harmaline), phenolic compounds, and enzyme-modulating metabolites.	Evaluate cytokine production, nitric oxide release, COX/LOX-related pathways, and cell viability to exclude false-positive anti-inflammatory effects.	[55,81,95,96]
**Antidiabetic**	β-carboline alkaloids, phenolics, and antioxidant constituents affecting carbohydrate metabolism.	Combine enzyme inhibition assays with cellular metabolic models, dose–response studies, bioavailability assessment, and toxicity evaluation.	[4,88,97,103]
**Anticancer**	β-carboline alkaloids (harmine, harmaline, harmalol) and structurally related derivatives.	Assess normal-cell selectivity, apoptosis, cell cycle regulation, target engagement, pharmacokinetics, and mechanism-based biomarkers rather than cytotoxicity alone.	[34,45,47,48,102,104]
**Agricultural bioactivity**	Allelopathic alkaloids, volatile phytochemicals, and secondary metabolites involved in plant defense.	Validate activity using seed-germination assays, pest and pathogen bioassays, non-target toxicity assessment, and field-relevant experimental conditions.	[23,24,105,106,107]

**Table 7:** Comparative toxicological characteristics of different *Peganum harmala* preparations.

Preparation	Main Toxic Constituents	Toxicological Evidence	Main Adverse Effects	References
Crude methanolic extract	Mixed phytochemicals and β-carbolines	LD_50_ > 3000 mg/kg; no significant subacute toxicity	Low acute toxicity	[95]
Oil extract	Lipophilic constituents	No observable toxicity up to 320 mg/kg	No major toxic effects reported	[95]
Purified β-carboline alkaloids (harmine/harmaline)	Harmine, harmaline	Higher neurotoxicity than crude extracts	Tremor, convulsions, hallucinations, respiratory depression, MAO inhibition	[1,44,108]
Essential oil (limited toxicological evidence)	Limited toxicological data available	Insufficient experimental evidence	Safety profile remains poorly characterized	[2,108]

**Table 8:** Safety-first evaluation framework for *Peganum harmala*: major toxicological concerns, risk drivers and minimum evidence required for translational development [35,36,53,54,96,98,99,100,101,109,110,113].

Safety Dimension	Principal Risk Driver	Minimum Evidence Required	Representative References
**Chemical potency**	Harmine, harmaline and other β-carboline alkaloids.	Quantitative chemical profiling, marker standardization, impurity analysis and batch-to-batch consistency.	[35,36,53,54,96]
**Exposure route**	Oral, inhalation (fumigation), topical and agricultural exposure.	Route-specific toxicological assessment, exposure estimation and pharmacokinetic evaluation.	[37,101,110]
**Drug interactions**	MAO inhibition, CYP modulation and transporter-mediated interactions.	Drug–drug interaction screening, mechanistic studies and contraindication assessment.	[36,53,54,96,100]
**Acute toxicity**	Neurological, cardiovascular and systemic adverse effects following high-dose exposure.	Acute toxicity studies, LD_50_ determination where appropriate, clinical case reports and mechanistic interpretation.	[98,99,109,110]
**Repeated exposure**	Potential cumulative toxicity and vulnerable populations.	Sub-chronic and chronic toxicity studies, biomarker monitoring and conservative exposure limits.	[98,99,100,101,109]
**Regulatory readiness**	Benefit–risk balance for the intended application.	Product specifications, contaminant control, stability studies and compliance with regulatory safety requirements.	[98,99,100,101,113]

**Table 9:** Translational roadmap for the development of *Peganum harmala*-derived products from experimental evidence to industrial applications [93,113,115,116,117,118,119,120,121,122,123,124,125].

Application Area	Opportunity	Primary Challenge	Recommended Development Pathway
**Drug discovery**	β-carboline alkaloids as lead structures for medicinal chemistry and target-based drug discovery	Safety profile, selectivity and pharmacokinetic limitations	Isolation, SAR optimization, ADMET evaluation and preclinical validation
**Cosmetic formulations**	Antioxidant, antimicrobial and anti-inflammatory ingredients for topical products	Dermal irritation, odor, pigmentation and alkaloid content	Low-alkaloid fractions, stability studies and dermatological safety assessment
**Food preservation**	Natural preservatives and active packaging systems	Sensory impact, migration behavior and consumer safety	Active packaging design, migration studies and food toxicology
**Agricultural bioprotection**	Botanical fungicides, insecticides and allelopathic bioherbicides	Non-target toxicity, environmental persistence and field efficacy	Field validation, ecological risk assessment and formulation optimization
**Advanced formulations**	Nanoemulsions, liposomes, hydrogels and controlled-release systems	Physical stability, oxidation and regulatory requirements	Quality-by-design formulation and marker standardization
**Industrial bioeconomy**	Sustainable valorization of arid-land biomass and high-value natural products	Supply consistency, standardization and scalable production	Cultivation strategies, chemotype selection and green extraction technologies

**Table 10:** Preliminary qualitative risk assessment for the development of *Peganum harmala*-derived products within a Quality-by-Design framework.

Factor	Risk Level	Potential Impact on Quality
Plant organ	High	Influences alkaloid and phenolic composition
Chemotype/geographical origin	High	Determines phytochemical profile and biological activity
Extraction solvent	High	Affects extraction selectivity and recovery of bioactive compounds
Extraction temperature	Medium	May alter thermolabile constituents
Drying conditions	Medium	Influences metabolite preservation before extraction
Storage conditions	Low	May affect long-term stability of volatile compounds

**Table 11:** Major research gaps, priority actions and expected outcomes for advancing *Peganum harmala* toward safe and sustainable industrial translation [111,112,114,125].

Research Gap	Why It Limits Progress	Priority Action	Expected Outcome
**Organ-specific chemotyping**	Seed-focused studies overlook the chemical diversity of leaves, roots and fruits.	Develop multi-organ GC-MS, LC-MS/MS and NMR chemotype maps across different geographical regions.	Predictive chemotype database supporting reproducible product development.
**Extraction standardization**	Limited comparability among extraction protocols prevents evidence-based technology selection.	Compare HD, SD, UAE, MAE and SC-CO_2_ using harmonized experimental designs (DoE).	Standardized extraction workflows optimized for yield, selectivity and sustainability.
**Mechanism-based bioactivity**	Many biological studies rely on crude extracts without clear chemical attribution.	Combine fractionation, marker quantification, molecular targets and recombination/synergy studies.	Mechanistically validated bioactivity with stronger translational relevance.
**Safety and toxicology**	Incomplete toxicological data limit pharmaceutical and industrial development.	Perform route-specific toxicity, pharmacokinetic and herb–drug interaction studies using standardized extracts.	Comprehensive safety profile supporting regulatory evaluation.
**Formulation development**	Stability, controlled release and shelf-life remain poorly investigated.	Develop nanoformulations, delivery systems and long-term stability studies under standardized conditions.	Stable, reproducible formulations suitable for pharmaceutical, cosmetic and food applications.
**Data integration and standardization**	Fragmented datasets reduce reproducibility and predictive modeling.	Integrate chemical, biological and metadata using open databases, molecular networking and AI-assisted chemoinformatics.	Reusable, interoperable datasets enabling predictive discovery and accelerated innovation.

## Data Availability

No new data were created or analyzed in this study. Data sharing is not applicable to this article.

## References

[1] Li S., Cheng X., Wang C. (2017). A Review on Traditional Uses, Phytochemistry, Pharmacology, Pharmacokinetics and Toxicology of the Genus Peganum. J. Ethnopharmacol..

[2] Li L.-N. (2024). *Peganum harmala* L.: A Review of Botany, Traditional Use, Phytochemistry, Pharmacology, Quality Marker, and Toxicity. Comb. Chem. High Throughput Screen..

[3] Dalli M., Fahd K., Azizi S., Gseyra N., Chaachouay N., Bussmann R.W., Bussmann R.W., Elachouri M., Kikvidze Z. (2024). *Peganum harmala* L. Zygophyllaceae. Ethnobotany of Northern Africa and Levant.

[4] İzol E., Turhan M., Yapıcı İ., Necip A., Abdullah Yılmaz M., Zengin G. (2025). Research of *Peganum harmala*: Phytochemical Content, Mineral Profile, Antioxidant, Antidiabetic, Anticholinergic Properties, and Molecular Docking. Chem. Biodivers..

[5] Zhu Y., Cheng F., Lu X., Ma X., Reyanggu A., Bakri M., Maiwulanjiang M. (2024). Profiling the Volatile Compounds of *Peganum harmala* L. Based on Multiple Sample Preparation Coupled with Gas Chromatography-Mass Spectrometry Analysis and Explored Its Antidepressants-like Activity. J. Chromatogr. B.

[6] Ghillace A., Chouitah O., Kiari F.Z., Fergoug Z., Daikh Z. (2025). Phytochemical Composition and Antimicrobial Activity of Essential Oil from the Leaves of *Peganum harmala* L. in Southern Algeria. Acta Biol. Slov..

[7] Mureed U., Tassaduq H., Zulfiqar A., Saddique Z. (2023). Identification of Chemical Composition of Essential Oil of Harmala Using Gc-Ms Analysis. Acta Chem. Malays. ACMY.

[8] Asadzadeh R., Abbasi N., Bahmani M. (2021). Extraction and Identification of Chemical Compounds of *Peganum harmala* L. Seed Essential Oil by HS-SPME and GC-MS Methods. Tradit. Integr. Med..

[9] Apostolico I., Aliberti L., Caputo L., De Feo V., Fratianni F., Nazzaro F., Souza L.F., Khadhr M. (2016). Chemical Composition, Antibacterial and Phytotoxic Activities of *Peganum harmala* Seed Essential Oils from Five Different Localities in Northern Africa. Molecules.

[10] Tepe M. (2026). Phytochemical Characterization and Antioxidant Potential of *Peganum harmala* L. Seeds: A Natural Source of Bioactive Compounds. Türk Doğa Fen Derg..

[11] Page M.J., McKenzie J.E., Bossuyt P.M., Boutron I., Hoffmann T.C., Mulrow C.D., Shamseer L., Tetzlaff J.M., Akl E.A., Brennan S.E. (2021). The PRISMA 2020 Statement: An Updated Guideline for Reporting Systematic Reviews. BMJ.

[12] Haddaway N.R., Macura B., Whaley P., Pullin A.S. (2018). ROSES RepOrting Standards for Systematic Evidence Syntheses: Pro Forma, Flow-Diagram and Descriptive Summary of the Plan and Conduct of Environmental Systematic Reviews and Systematic Maps. Environ. Evid..

[13] Bechis G., Minteguiaga M.A., Sgorbini B., Marengo A., Rubiolo P., Cagliero C. (2023). Make the Quality Control of Essential Oils Greener: Fast Enantioselective GC-MS Analysis of Sweet and Bitter Orange as a Case Study. Molecules.

[14] Yang H., Huang X., Yang M., Zhang X., Tang F., Gao B., Gong M., Liang Y., Liu Y., Qian X. (2024). Advanced Analytical Techniques for Authenticity Identification and Quality Evaluation in Essential Oils: A Review. Food Chem..

[15] Bodea I.M., Garre Pérez A., Cătunescu G.M., Palop A. (2025). A Review on Microwave and Ultrasound-Assisted Extractions of Essential Oil from Orange Peel Waste. Food Bioprocess Technol..

[16] El Ahmadi K., El Allaoui H., El Abdouni A., Bouhrim M., Eto B., Dira I., Shahat A.A., Herqash R.N., Haboubi K., El Bastrioui M. (2024). A Bibliometric Analysis of the Supercritical CO2 Extraction of Essential Oils from Aromatic and Medicinal Plants: Trends and Perspectives. Horticulturae.

[17] El-Saadony M.T., Saad A.M., Mohammed D.M., Alkafaas S.S., Abd El-Mageed T.A., Fahmy M.A., Ezzat Ahmed A., Algopishi U.B., Abu-Elsaoud A.M., Mosa W.F. (2025). Plant Bioactive Compounds: Extraction, Biological Activities, Immunological, Nutritional Aspects, Food Application, and Human Health Benefits—A Comprehensive Review. Front. Nutr..

[18] Kırkıncı S., Gercek Y.C., Baştürk F.N., Yıldırım N., Gıdık B., Bayram N.E. (2024). Evaluation of Lavender Essential Oils and By-Products Using Microwave Hydrodistillation and Conventional Hydrodistillation. Sci. Rep..

[19] Rostampour M., Yousefian M., Yari R. (2026). The Impact of *Peganum harmala* L. Invasion on the Density and Species Diversity of Native Plants. J. Rangel. Sci..

[20] Li Y., Tian H., He Q., Geng Z., Yan S., Tuniyazi G., Bai X. (2021). Investigation of the Geographical Environment Impact on the Chemical Components of *Peganum harmala* L. Through a Combined Analytical Method. ACS Omega.

[21] Shao H., Huang X., Zhang Y., Zhang C. (2013). Main Alkaloids of *Peganum harmala* L. and Their Different Effects on Dicot and Monocot Crops. Molecules.

[22] Shi K., Shao H. (2022). Changes in the Soil Fungal Community Mediated by a *Peganum harmala* Allelochemical. Front. Microbiol..

[23] Vantarová K.H., Eliáš P., Jiménez-Ruiz J., Tokarska-Guzik B., Cires E. (2023). Biological Invasions in the Twenty-First Century: A Global Risk. Biologia.

[24] Bobul’ská L., Demková L., Pinčáková G., Lošák T. (2025). Alien Plant Invasion: Are They Strictly Nature’s Enemy and How Can We Use Their Supremacy?. Land.

[25] Gammoudi T., Besser H., Chaieb A., Abdelli F., Mahjoubi A., Nardi F. (2025). Aromatic and Medicinal Plant (AMP) Valorization via a Farmer-Centric Approach for the Sustainable Development of Climate-Challenged Areas Affected by Rural Exodus (Southeastern Tunisia). Sustainability.

[26] Li S., Yan N., Tanveer M., Zhao Z., Jiang L., Wang H. (2023). Seed Germination Ecology of the Medicinal Plant *Peganum harmala* (Zygophyllaceae). Plants.

[27] Sardarova A. (2025). Anatomical Diagnostic Indicators of Adaptation to Ecological Conditions in the Vegetative and Generative Organs of *Peganum harmala* (Nitrariaceae). Biosyst. Divers..

[28] Boulkroune M., Dekkiche B., Abassi Y. (2023). *Peganum harmala* Seeds as a Sustainable Source of Pickling Inhibitor for Zinc: Anti-Oxidant Properties, Adsorption Behaviour, Electrochemical Studies, and Synergistic Effect. Anal. Bioanal. Electrochem..

[29] Bhuiyan M.N.I., Rahman M.S., Rahman M.M., Saha B.K. (2026). Artificial Intelligence and Essential Oils for Advanced Food Preservation. Discov. Food.

[30] Li S.-G., Xiang L.-L., Zhang J.-J., Ling L.-X., Liu Q.-H., Zhang W., Hua H.-M., Hu X., Huang X.-Z. (2026). Three New Alkaloids from the Roots of *Peganum harmala*. J. Asian Nat. Prod. Res..

[31] Shengge L.I., Zhang Q., Yuetong W., Bin L.I.N., Dahong L.I., Huiming H.U.A., Xu H.U. (2024). β-Carboline Alkaloids from the Roots of *Peganum harmala* L.. Chin. J. Nat. Med..

[32] Yang Y., Cheng X., Liu W., Han Z., Chou G., Wang Y., Sun D., Wang Z., Wang C. (2016). Peganumine BI and Two Enantiomers: New Alkaloids from the Seeds of *Peganum harmala* Linn. and Their Potential Cytotoxicity and Cholinesterase Inhibitory Activities. RSC Adv..

[33] Wang C., Zhang Z., Wang Y., He X. (2015). Cytotoxic Indole Alkaloids against Human Leukemia Cell Lines from the Toxic Plant *Peganum harmala*. Toxins.

[34] Timbilla A.A., Vrabec R., Havelek R., Rezacova M., Chlebek J., Blunden G., Cahlikova L. (2025). The Anticancer Properties of Harmine and Its Derivatives. Phytochem. Rev..

[35] Alonso P., Fernández-Pastor S., Guerrero A. (2024). Application of Cinnamon Essential Oil in Active Food Packaging: A Review. Appl. Sci..

[36] Vitale G.A., Geibel C., Minda V., Wang M., Aron A.T., Petras D. (2024). Connecting Metabolome and Phenotype: Recent Advances in Functional Metabolomics Tools for the Identification of Bioactive Natural Products. Nat. Prod. Rep..

[37] Araneda J.F., Leclerc M.C., Riegel S.D. (2026). Quality Control Assays of Essential Oils Using Benchtop NMR Spectroscopy: Quantification of Key Terpenes, Terpenoids, and Aldehydes Using an Internal Calibrant Approach. Magn. Reson. Chem..

[38] Ben Miri Y. (2025). Essential Oils: Chemical Composition and Diverse Biological Activities: A Comprehensive Review. Nat. Prod. Commun..

[39] Baldini E., Cardarelli S., Campese A.F., Lori E., Fallahi P., Virili C., Forte F., Pironi D., Di Matteo F.M., Palumbo P. (2024). Evaluation of the Therapeutic Effects of Harmine on Anaplastic Thyroid Cancer Cells. Int. J. Mol. Sci..

[40] Egger K., Aicher H.D., Cumming P., Scheidegger M. (2024). Neurobiological Research on N,N-Dimethyltryptamine (DMT) and Its Potentiation by Monoamine Oxidase (MAO) Inhibition: From Ayahuasca to Synthetic Combinations of DMT and MAO Inhibitors. Cell. Mol. Life Sci..

[41] Benny F., Kumar S., Jayan J., Abdelgawad M.A., Ghoneim M.M., Kumar A., Manoharan A., Susan R., Sudevan S.T., Mathew B. (2023). Review of B-carboline and Its Derivatives as Selective MAO-A Inhibitors. Arch. Pharm..

[42] Abbas M.W., Hussain M., Qamar M., Ali S., Shafiq Z., Wilairatana P., Mubarak M.S. (2021). Antioxidant and Anti-Inflammatory Effects of *Peganum harmala* Extracts: An In Vitro and In Vivo Study. Molecules.

[43] Faridi P., Ghasemi Y., Mohagheghzadeh A. (2013). Chemical Composition of *Peganum harmala* Smoke and Volatile Oil. J. Essent. Oil Bear. Plants.

[44] Doskaliev A., Seidakhmetova R.B., Tutai D.S., Goldaeva K., Surov V.K., Adekenov S.M. (2021). Alkaloids of *Peganum harmala* L. and Their Pharmacological Activity. Open Access Maced. J. Med. Sci..

[45] Begum S., Zubair F., Nawazish Ali S., Shaheen Siddiqui B. (2009). An Efficient, Mild and Solvent-Free Synthesis of Benzene Ring Acylated Harmalines. Molecules.

[46] Jalali A., Dabaghian F., Zarshenas M.M. (2021). Alkaloids of *Peganum harmala*: Anticancer Biomarkers with Promising Outcomes. Curr. Pharm. Des..

[47] Wang Y., Wang Y., Zhang Z., Xu K., Fang Q., Wu X., Ma S. (2025). Molecular Networking: An Efficient Tool for Discovering and Identifying Natural Products. J. Pharm. Biomed. Anal..

[48] Cho H.M., Boccia E., Rajwani R., O’Connor R.D., Boshoff H., Barry C., Bifulco G., Bewley C.A. (2025). A Systematic Approach to Discover New Natural Product Scaffolds Using Database-Derived Relative Mass Spectral Defects and Molecular Networking. JACS Au.

[49] Kartal M., Altun M.L., Kurucu S. (2003). HPLC Method for the Analysis of Harmol, Harmalol, Harmine and Harmaline in the Seeds of *Peganum harmala* L.. J. Pharm. Biomed. Anal..

[50] Sanches C.G.S., Bernardi L.S., Siqueira G.R., De Oliveira P.R. (2026). High-Performance Liquid Chromatography Applied to Essential Oils: Analytical Approaches in the Literature. Chem. Afr..

[51] Salimizadeh Z., Enferadi S.T., Majidizadeh T., Mahjoubi F. (2024). Cytotoxicity of Alkaloids Isolated from *Peganum harmala* Seeds on HCT116 Human Colon Cancer Cells. Mol. Biol. Rep..

[52] Alguacil M.C., Umeki K., Gaidukovs S., Barkāne A., You S., Joffe R. (2024). The Impact of Thermal Treatment Parameters on the Preservation of Carbon Fiber Mechanical Properties after Reclamation. Curr. Res. Green Sustain. Chem..

[53] Jiang N., Chen L., Li J., Li W., Jiang S. (2023). Lethal and Sublethal Toxicity of Beta-Carboline Alkaloids from *Peganum harmala* (L.) against Aedes Albopictus Larvae (Diptera: Culicidae). Toxics.

[54] Yu L., Shen N., Ren J., Xin H., Cui Y. (2025). Resource Distribution, Pharmacological Activity, Toxicology and Clinical Drugs of β-Carboline Alkaloids: An Updated and Systematic Review. Fitoterapia.

[55] Benarous K., Bombarda I., Iriepa I., Moraleda I., Gaetan H., Linani A., Tahri D., Sebaa M., Yousfi M. (2015). Harmaline and Hispidin from *Peganum harmala* and Inonotus Hispidus with Binding Affinity to Candida Rugosa Lipase: In Silico and In Vitro Studies. Bioorg. Chem..

[56] El Ahmadi K., Haboubi K., El Abdouni A., El Alaoui H., Dira I., El Bastrioui M., Dimane F., El Hammoudani Y. (2024). Evaluation of a New Process for Extracting Essential Oil from Aromatic, Medicinal, and Pharmaceutical Plants. Proceedings of the E3S Web of Conferences.

[57] Muddather H.F., Nasibova T., Szebeni G.J., Gémes N., Bózsity N., Minorics R., Garayev E., Orhan I.E., Herbette G., Schelz Z. (2025). Potential Anticancer Effects of *Peganum harmala* in Human Papillomavirus-Related Cervical and Head and Neck Cancer Cells. Front. Pharmacol..

[58] Gedikoğlu A., Öztürk H.İ., Özçoban A. (2024). Analysis of the Chemical Composition, Antimicrobial, and Antioxidant Qualities of Microwave and Supercritical CO_2_-Extracted Lavender Essential Oils Cultivated in a Hyperarid Region of Türkiye. Molecules.

[59] Li S., Zhang Y., Deng G., Wang Y., Qi S., Cheng X., Ma Y., Xie Y., Wang C. (2017). Exposure Characteristics of the Analogous β-Carboline Alkaloids Harmaline and Harmine Based on the Efflux Transporter of Multidrug Resistance Protein 2. Front. Pharmacol..

[60] Chaurasiya N.D., Leon F., Muhammad I., Tekwani B.L. (2022). Natural Products Inhibitors of Monoamine Oxidases—Potential New Drug Leads for Neuroprotection, Neurological Disorders, and Neuroblastoma. Molecules.

[61] Krajewska A., Azeez G., Ebadollahi A., Kalemba D., Synowiec A. (2026). Carvone-Rich Essential Oils and Their Agrobiological Interactions: A Review. Molecules.

[62] Morehouse N.J., Clark T.N., McMann E.J., van Santen J.A., Haeckl F.J., Gray C.A., Linington R.G. (2023). Annotation of Natural Product Compound Families Using Molecular Networking Topology and Structural Similarity Fingerprinting. Nat. Commun..

[63] Ciobotaru G.V., Goje I.-D., Dehelean C.A., Danciu C., Magyari-Pavel I.Z., Moacă E.-A., Muntean D., Imbrea I.M., Sărățeanu V., Pop G. (2025). Analysis of the Antioxidant and Antimicrobial Activity, Cytotoxic, and Anti-Migratory Properties of the Essential Oils Obtained from Cultivated Medicinal Lamiaceae Species. Plants.

[64] Erfaninejad M., Aboualigalehdari E., Fatahinia M. (2023). Effect of *Peganum harmala* Extract on Biofilm and Involved Gene Expression in Biofilm Production of *Candida albicans*. Jundishapur J. Microbiol..

[65] Djermane N., Brahmi M., Adli D.E., Gliozzi M., Musolino V., Khellaf R., Erenler R., Arhab R., Garzoli S. (2025). Chemical Composition, Antioxidant, Antimicrobial, Antidiabetic, and Butyrylcholinesterase Inhibitory Activities in Vitro of the Essential Oil and Crude Extracts of the Aerial Parts of *Thymus ciliatus*. Chem. Biodivers..

[66] Trovato E., Balcerzak L., Vidal C., Strub D.J., Fakhry H.A., Dugo P., Mondello L. (2024). Gas Chromatographic, Sensory Profile and Biological Properties Evaluation of Egyptian *Jasminum grandiflorum* Essential Oil Produced Industrially by Steam Distillation. J. Essent. Oil Res..

[67] Achoukhi I., El Hammoudani Y., Dimane F., Haboubi K., Bourjila A., Haboubi C., Benaissa C., Elabdouni A., Faiz H. (2023). Investigating Microplastics in the Mediterranean Coastal Areas–Case Study of Al-Hoceima Bay, Morocco. J. Ecol. Eng..

[68] Benabderrahmane W., Fadel H., Sekhara I., Mennai I., Kadi I.E., Helal M., Sami R., Abo-Dief H.M., Bedaiwi R.I., Alanazi M.A. (2024). GC-MS Analysis, Phytochemical Composition of *Hertia cheirifolia* L. Essential Oil with Pharmacological Assessments: Antioxidant, Antibacterial, and Antifungal Activities. RSC Adv..

[69] Nouioura G., El Fadili M., El Hachlafi N., Maache S., Mssillou I., Abuelizz H.A., Lafdil F.Z., Er-Rahmani S., Lyoussi B., Derwich E. (2024). *Coriandrum sativum* L., Essential Oil as a Promising Source of Bioactive Compounds with GC/MS, Antioxidant, Antimicrobial Activities: In Vitro and in Silico Predictions. Front. Chem..

[70] Li S.-P., Wang Y.-W., Qi S.-L., Zhang Y.-P., Deng G., Ding W.-Z., Ma C., Lin Q.-Y., Guan H.-D., Liu W. (2018). Analogous β-Carboline Alkaloids Harmaline and Harmine Ameliorate Scopolamine-Induced Cognition Dysfunction by Attenuating Acetylcholinesterase Activity, Oxidative Stress, and Inflammation in Mice. Front. Pharmacol..

[71] Ravaglia L.M., de Oliveira P.D., Holzgrabe U., Alcantara G.B. (2024). qNMR in Natural Products: Practical Approaches. What Nobody Tells You before Starting Your qNMR Study!. Front. Nat. Prod..

[72] Ayllón-Gutiérrez R., Díaz-Rubio L., Montaño-Soto M., Haro-Vázquez M.d.P., Córdova-Guerrero I. (2024). Applications of Plant Essential Oils in Pest Control and Their Encapsulation for Controlled Release: A Review. Agriculture.

[73] Deveci E., Altınok B.Y., Tel-çayan G. (2025). The Preliminary Study on Phytochemical Profile and Antioxidant and Cytotoxic Activities of Harmal (*Peganum harmala* L.). Commagene J. Biol..

[74] Rout R.K., Kukde R.B., Sivamma P., Kumar A., Prakash R., Srivastav P.P., Srivastava B., Karunanithi S. (2025). Methodology for Extraction of Essential Oil Using Ultrasonic-Microwave-Assisted Hydrodistillation. Essential Oil Extraction from Food By-Products.

[75] Fadil M.E., Fakhouri K.E., Boulamtat R., Henkrar F., Ramdani C., Bargui K.E., Oubayoucef A., Drissi B., Taarji N., Bouhssini M.E. (2023). *Lavandula angustifolia* Mill. Essential Oil Exhibits Distinct Insecticidal Activities Against Pea Leaf Weevil Adults on Faba Bean under Laboratory and Growth Chamber Conditions. ACS Agric. Sci. Technol..

[76] Soulaimani B. (2025). Comprehensive Review of the Combined Antimicrobial Activity of Essential Oil Mixtures and Synergism with Conventional Antimicrobials. Nat. Prod. Commun..

[77] Ramundi V., Zdouc M.M., Donati E., Van Der Hooft J.J.J., Cimini S., Righetti L. (2025). Non-Targeted Metabolomics-Based Molecular Networking Enables the Chemical Characterization of Rumex Sanguineus, a Wild Edible Plant. Metabolomics.

[78] Ali S., Ekbbal R., Salar S., Yasheshwar, Ali S.A., Jaiswal A.K., Singh M., Yadav D.K., Kumar S., Gaurav (2023). Quality Standards and Pharmacological Interventions of Natural Oils: Current Scenario and Future Perspectives. ACS Omega.

[79] Zhang M., Otsuki K., Li W. (2023). Molecular Networking as a Natural Products Discovery Strategy. Acta Mater. Med..

[80] De Farias P.M., De Sousa R.V., Maniglia B.C., Pascall M., Matthes J., Sadzik A., Schmid M., Fai A.E.C. (2025). Biobased Food Packaging Systems Functionalized with Essential Oil via Pickering Emulsion: Advantages, Challenges, and Current Applications. ACS Omega.

[81] Ables J.L., Israel L., Wood O., Govindarajulu U., Fremont R.T., Banerjee R., Liu H., Cohen J., Wang P., Kumar K. (2024). A Phase 1 Single Ascending Dose Study of Pure Oral Harmine in Healthy Volunteers. J. Psychopharmacol..

[82] El Ahmadi K., El Allaoui H., El Abdouni A., Bouhrim M., Etu B., Dira I., Shahat A., Herqash R.N., Haboubi K., El Bastrioui M. (2025). Bibliometric and Comparative Analysis of Research on Essential Oils and Aromatic and Medicinal Plants in Morocco: Positioning and Perspectives in the World. J. King Saud Univ..

[83] Liu T., Liu J., Mao X., Jiang X., Zhao Y., Qian Y. (2023). Rapid and Portable Detection of Hg and Cd in Grain Samples Based on Novel Catalytic Pyrolysis Composite Trap Coupled with Miniature Atomic Absorption Spectrometry. Foods.

[84] El Ahmadi K., Haboubi K., El Allaoui H., Nasr F.A., Lamsiah M., Hayoune A., El Hammoudani Y., Bouhrim M., Al-Zharani M., Qurtam A.A. (2026). Environmental Determinants of Cannabinoid, Terpene and Safety Profiles in *Cannabis sativa* L. Cultivation in Northern Morocco. Ind. Crops Prod..

[85] De Luca M., Tripiciano C., D’Amore C., Ciofi Degli Atti M.L., Romani L., Pagano F., Zama D., Garazzino S., Nicolini G., Bosis S. (2025). Current Clinical Practice on the Management of Invasive Streptococcus Pyogenes Infections in Children: A Survey-Based Study. Antibiotics.

[86] Noshirvani N. (2024). Essential Oils as Natural Food Preservatives: Special Emphasis on Antimicrobial and Antioxidant Activities. J. Food Qual..

[87] Mohammadi F., Karimi E., Homayouni-Tabrizi M., Oskoueian E. (2024). Nanoparticle-Based Delivery of Harmine: A Comprehensive Study on Synthesis, Characterization, Anticancer Activity, Angiogenesis and Toxicity Evaluation. Heliyon.

[88] Alossaimi M.A., Abdel Bar F.M., Elekhnawy E., ElNaggar M.H. (2025). Antibacterial and Antiviral Potential of Harmalacidine Hydrochloride, a β-Carboline Alkaloid, against Respiratory Tract Pathogens: Staphylococcus Aureus and H1N1 Influenza Virus. PLoS ONE.

[89] Wu G., Wang W., Li F., Xu C., Zhou Y., Li Z., Liu B., Shao L., Chen D., Bai S. (2024). Design, Synthesis and Biological Activity Evaluation of β-Carboline Derivatives Containing Nitrogen Heterocycles. Molecules.

[90] Liu Q., Yang C., Qi J., Shen Q., Ye M., Li H., Zhang L. (2025). Bioactivities and Structure–Activity Relationships of Harmine and Its Derivatives: A Review. Chem. Biodivers..

[91] Hu Y., Yu X., Yang L., Xue G., Wei Q., Han Z., Chen H. (2024). Research Progress on the Antitumor Effects of Harmine. Front. Oncol..

[92] Bianco L.S., Nicoliche T., Corrêa-Neto N.F., Lanaro R., Caetano A.L., Prado C.M., Ureshino R.P., Tibério I.d.F.L.C., Righetti R.F., Stilhano R.S. (2026). Anti-Inflammatory Effects of Banisteriopsis Caapi and Beta-Carbolines in Neuronal Cells: Potential Implications for Neuro-COVID. Front. Pharmacol..

[93] Hernández-Bolaños E., Sánchez-Retuerta V., Matías-Hernández L., Cuyas L. (2025). Promising Applications on the Use of Medicinal and Aromatic Plants in Agriculture. Discov. Agric..

[94] Yu Y., Kuballa T., Lachenmeier D.W. (2024). NMR Analysis of Pulegone in Food Products. Appl. Sci..

[95] Liu W., Wang Y., He D., Li S., Zhu Y., Jiang B., Cheng X., Wang Z., Wang C. (2015). Antitussive, Expectorant, and Bronchodilating Effects of Quinazoline Alkaloids (±)-Vasicine, Deoxyvasicine, and (±)-Vasicinone from Aerial Parts of *Peganum harmala* L.. Phytomedicine.

[96] Iskandar K., Ahmed N., Paudyal N., Ruiz Alvarez M.-J., Balasubramani S.P., Saadeh D., Ullah Baig S., Sami H., Hammoudi Halat D., Pavlović N. (2025). Essential Oils as Antimicrobial Agents against WHO Priority Bacterial Pathogens: A Strategic Review of In Vitro Clinical Efficacy, Innovations and Research Gaps. Antibiotics.

[97] Ostadkarampour M., Putnins E.E. (2021). Monoamine Oxidase Inhibitors: A Review of Their Anti-Inflammatory Therapeutic Potential and Mechanisms of Action. Front. Pharmacol..

[98] Caesar L.K., Montaser R., Keller N.P., Kelleher N.L. (2021). Metabolomics and Genomics in Natural Products Research: Complementary Tools for Targeting New Chemical Entities. Nat. Prod. Rep..

[99] Newman D.J., Cragg G.M. (2020). Natural Products as Sources of New Drugs over the Nearly Four Decades from 01/1981 to 09/2019. J. Nat. Prod..

[100] Lee J., Hyun C.-G. (2023). Natural Products for Cosmetic Applications. Molecules.

[101] Lu Y.-S., Qiu J., Mu X.-Y., Qian Y.-Z., Chen L. (2024). Levels, Toxic Effects, and Risk Assessment of Pyrrolizidine Alkaloids in Foods: A Review. Foods.

[102] Dhotre I. (2025). A Comprehensive Review on Progression and Innovations in Microwave Assisted Extraction Technology for Essential Oils. J. Chem. Technol. Biotechnol..

[103] Cadena-Cadena F., Arias-Moscoso J.L., Argentel-Martínez L., Torres Velazquez J.R., Cuevas-Acuña D.A., Buitimea Cantua N.E., Concha-Frías B. (2025). Effect of Ultrasonic Pretreatment on the Extraction Process of Essential Oils from Grapefruit (*Citrus paradisi*) by-Products. BioTech.

[104] Pakkir Shah A.K., Walter A., Ottosson F., Russo F., Navarro-Diaz M., Boldt J., Kalinski J.-C.J., Kontou E.E., Elofson J., Polyzois A. (2025). Statistical Analysis of Feature-Based Molecular Networking Results from Non-Targeted Metabolomics Data. Nat. Protoc..

[105] Andrade M.A., Barbosa C.H., Ribeiro-Santos R., Tomé S., Fernando A.L., Silva A.S., Vilarinho F. (2025). Emerging Trends in Active Packaging for Food: A Six-Year Review. Foods.

[106] El Allaoui H., El Ahmadi K., Elabdouni A., Bouhrim M., Eto B., Hussein A.A.S., Herqash R.N., Haboubi K., El Bastrioui M., El Hammoudani Y. (2025). Selection of Essential Oil Extraction Methods Using the Analytic Hierarchy Process (AHP) and the Preference Ranking Organization Method for Enrichment Evaluations (PROMETHEE) Approaches. J. King Saud Univ..

[107] Joshi H., Bhushan S., Dimri T., Sharma D., Sak K., Chauhan A., Chauhan R., Haque S., Ahmad F., Kumar M. (2025). Anti-Tumor Potential of Harmine and Its Derivatives: Recent Trends and Advancements. Discov. Oncol..

[108] Chelu M. (2024). Hydrogels with Essential Oils: Recent Advances in Designs and Applications. Gels.

[109] Moshiri M., Etemad L., Javidi S., Alizadeh A. (2013). *Peganum harmala* Intoxication, a Case Report. Avicenna J. Phytomed..

[110] Hefter H., Schomaecker I., Schomaecker M., Ürer B., Brauns R., Rosenthal D., Albrecht P., Samadzadeh S. (2023). Lessons about Botulinum Toxin A Therapy from Cervical Dystonia Patients Drawing the Course of Disease: A Pilot Study. Toxins.

[111] Wang D., Chu F., Wei H., Zhang J., Zhai H. (2025). Recent Advances in the Synergistic Application of Essential Oils and Emerging Technologies for Fresh Food Preservation: A Comprehensive Review. Compr. Rev. Food Sci. Food Saf..

[112] Sodaeizadeh H., Rafieiolhossaini M., Van Damme P. (2010). Herbicidal Activity of a Medicinal Plant, *Peganum harmala* L., and Decomposition Dynamics of Its Phytotoxins in the Soil. Ind. Crops Prod..

[113] Manful M.E., Ahmed L., Barry-Ryan C. (2024). Cosmetic Formulations from Natural Sources: Safety Considerations and Legislative Frameworks in the European Union. Cosmetics.

[114] Haboubi K., El Abdouni A., El Hammoudani Y., Dimane F., Haboubi C. (2023). Estimating Biogas Production in the Controlled Landfill of Fez (Morocco) Using The Land-Gem Model. Environ. Eng. Manag. J. EEMJ.

[115] Haboubi C., Barhdadi E.H., Haboubi K., Hammoudani Y.E., Sadoune Z., El Abdouni A., Dimane F. (2024). Characterization of the Mechanical Behavior of Hemp-Clay Composites. Adv. Sci. Technol. Res. J..

[116] Tavares L., Noreña C.P.Z., Barros H.L., Smaoui S., Lima P.S., de Oliveira M.M. (2022). Rheological and Structural Trends on Encapsulation of Bioactive Compounds of Essential Oils: A Global Systematic Review of Recent Research. Food Hydrocoll..

[117] De Carvalho A.P.A., Weitz D.A., Conte-Junior C.A. (2026). Green Oil-in-Water Nanoemulsions for Delivery of Phytochemicals with Pesticidal Activity for Sustainable Food Production and Safety. Compr. Rev. Food Sci. Food Saf..

[118] Mohammed D.M., Salem M.B., Elzallat M., Alkafaas S.S., Salem H.M., Alnadari F., Ibrahim E.H., Elkelish A., Negm S.H., Saad A.M. (2026). Essential Oils: Antimicrobial, Antioxidant Properties, and Their Applications in Food Preservation—A Comprehensive Review. Phytochem. Rev..

[119] Zhu R., Morkos B., Liu L. (2026). Therapeutic Potentials and Encapsulation Strategies of Essential Oils. Processes.

[120] Khan S., Abdo A.A., Shu Y., Zhang Z., Liang T. (2023). The Extraction and Impact of Essential Oils on Bioactive Films and Food Preservation, with Emphasis on Antioxidant and Antibacterial Activities—A Review. Foods.

[121] Abdalla S., Aroua M.K., Gew L.T. (2024). A Comprehensive Review of Plant-Based Cosmetic Oils (Virgin Coconut Oil, Olive Oil, Argan Oil, and Jojoba Oil): Chemical and Biological Properties and Their Cosmeceutical Applications. ACS Omega.

[122] Michailidou F. (2023). The Scent of Change: Sustainable Fragrances Through Industrial Biotechnology. ChemBioChem.

[123] Atanasov A.G., Zotchev S.B., Dirsch V.M., Supuran C.T., International Natural Product Sciences Taskforce (2021). Natural Products in Drug Discovery: Advances and Opportunities. Nat. Rev. Drug Discov..

[124] Akl M.M., Ahmed A. (2025). Exploring *Peganum harmala* as a Natural Alternative to Semaglutide: A Novel Approach to Glucagon-like Peptide-1 Stimulation and Insulin Sensitization. Innov. Med. Omics.

[125] White E., Kennedy T., Ruffell S., Perkins D., Sarris J. (2024). Ayahuasca and Dimethyltryptamine Adverse Events and Toxicity Analysis: A Systematic Thematic Review. Int. J. Toxicol..

[126] Moradi M.T., Bagherian Khouzani N., Rafieian A., Asadi-Samani M., Khosravi Farsani M.R., Mousiany N. (2026). Antiproliferative and apoptosis-inducing effects of *Peganum harmala* L. seed extracts on gastric adenocarcinoma. J. Herbmed. Pharmacol..

[127] Chen L., Mulder P.P., Louisse J., Peijnenburg A., Wesseling S., Rietjens I.M. (2017). Risk Assessment for Pyrrolizidine Alkaloids Detected in (Herbal) Teas and Plant Food Supplements. Regul. Toxicol. Pharmacol..

[128] Merino J.J., Durán A.G., Chinchilla N., Macías F.A. (2025). Biological Activities of Hydroxyanthracene Derivatives (HADs) from Aloe Species and Their Potential Uses. Phytochem. Rev..

[129] Cimino C., Maurel O.M., Musumeci T., Bonaccorso A., Drago F., Souto E.M.B., Pignatello R., Carbone C. (2021). Essential Oils: Pharmaceutical Applications and Encapsulation Strategies into Lipid-Based Delivery Systems. Pharmaceutics.

[130] Elgadir M.A., Alhudhaibi A.M., Abdallah E.M., Adiletta G. (2025). Plant-Based Preservation of Meat: A Critical Narrative Review of Bioactive Extracts, Essential Oils, and next-Generation Delivery Systems. Front. Sustain. Food Syst..

